# Single-cell ionomes of terrestrial cryosphere algae

**DOI:** 10.1038/s43247-026-03679-6

**Published:** 2026-06-26

**Authors:** Silvana R. Oliveira, Helen K. Feord, Cícero A. Lopes Júnior, Ravi Sven Peters, Liane G. Benning, Björn Meermann

**Affiliations:** 1 Federal Institute for Materials Research and Testing (BAM), Division 1.1–Inorganic Trace Analysis (ITALab), Berlin, Germany; 2 Department of Clinical Analyses, Toxicology and Food Sciences, School of Pharmaceutical Sciences of Ribeirão Preto, University of São Paulo (USP), Ribeirão Preto, Brazil; 3 GFZ Helmholtz Center for Geosciences, Potsdam, Germany; 4 Grupo de Estudos em Bioanalítica–GEBIO, Department of Chemistry, Federal University of Piauí, Teresina, Brazil; 5 Department of Earth Sciences, Freie Universität Berlin, Berlin, Germany

**Keywords:** Cryospheric science, Environmental sciences, Microbiology

## Abstract

Algae from the Viridiplantae lineage grow on glaciers and semi-permanent snow patches globally. These algal taxa have adapted to extreme environmental conditions, such as freezing temperatures, high light, and oligotrophic nutrient availability. However, how cryosphere algae balance their cellular nutrients to deal with these conditions is not fully known. To address this knowledge gap, we used single-cell inductively coupled plasma-mass spectrometry to quantify the single-cell ionomes (Phosphorus, Magnesium, Calcium, Copper, Iron, Manganese, and Zinc) of cryosphere chlorophyte algae, *Microglena sp*., *Raphidonema sempervirens*, and *Deuterostichococcus sp*., and compared them to mesophile chlorophyte algae, *Acutodesmus obliquus* and *Chlamydomonas reinhardtii*. We validated our results through mass spectrometric analyses of digested cultures. When corrected to average cell biovolume, cryosphere algae had lower Phosphorus, Magnesium, and Calcium, consistent with slow cellular metabolism adapted to cold life. Under Phosphorus-starvation, *Raphidonema* showed no impact of extracellular Phosphorus loss on its single-cell ionome, however, *Microglena* had a correlative loss of Phosphorus and Magnesium, and an increase in Copper, indicating Phosphorus mobilisation *via* polyphosphate hydrolysis. Our results provide a comprehensive insight into the single-cell ionomes of cryosphere and mesophile chlorophyte algae and reveal insights into nutrient homeostasis in algal cells adapted to the cryosphere.

## Abstract (web summary)

Cryosphere algae have low phosphorus, calcium, and magnesium, which is consistent with slow cellular metabolism adapted to cold life, according to single-cell inductively coupled plasma-mass spectrometry used to quantify the single-cell ionomes.

## Introduction

The terrestrial cryosphere, which includes glaciers and semi-temporary snow patches, is host to thriving microbial communities^1^. Eukaryotic algae are often the primary producers in these ecosystems. These algae belong to the Viridiplantae lineage, with streptophyte algae of the *Ancylonema* genus blooming on glacier ice, and chlorophyte algae of the Chlorophyceae class, for example *Sanguina* spp., *Microglena* spp., and *Chloromonas* spp., blooming on snow. These Chlorophyceae algae exist in vegetative stages (as green snow algae) or as zygotes, rich in secondary carotenoids (as red snow algae). Other algae also exist in these environments in low cell abundance, such as chlorophyte algae of the Trebouxiophyceae class, for example *Raphidonema* spp. and *Deuterostichococcus* spp^2,3^. All algal taxa living on glacier ice and snow surfaces have adapted to extreme environmental conditions comparative to their mesophilic cousins. These conditions include low to freezing air and substrate temperatures, low liquid water availability, and high photosynthetically active and UV radiation. Additionally, low nutrient availability (oligotrophy) is often characteristic of terrestrial cryosphere environments^4–8^. Nutrient availability is dynamic on glaciers, changing temporally throughout the melt season as well as spatially, including between sub-habitats that are close to each other^4,9–13^. Among other factors^7,14^, differences in nutrient availability in terrestrial cryosphere environments have also been found to be associated with changes in microbiome community composition, comprising the dominant algal cell type. In particular, green snow algae blooms are generally associated with higher nutrient availability compared to red snow algae blooms^12,15^.

Adaptation to low and variable nutrient availability in glacier ice and snow algae happens at a taxa-specific cellular level, with previous work generally focused on N and P^3,5–8,16–19^ as limiting nutrients. If a specific nutrient concentration in the substrate is low or maybe even limited, algal cells must regulate their metabolic pathways to compensate for scarce nutrient availability to survive. A low cellular macro-nutrient content and elevated C:N and C:P ratios have been found to be common for snow algae and glacier ice algae, possibly reflecting adaptations to specific oligotrophic surface snow and ice environments^17,18^. In certain species of snow algae (for example *Chloromonas*), N-limitation contributes to the formation of carotenoid- and lipid-rich cysts stages^3^. On the other hand, glacier ice algae were shown to store P, essential for ATP, DNA and RNA synthesis, as polyphosphate^18^. In terms of trace metal nutrition, there are indications of the importance of Fe for snow algae culture growth^20,21^ and glacier ice algae pigmentation^22^, as well as the uptake of Fe and Mn in environmental snow algae samples^23^. However, no information about the full elemental profiles of cryosphere algae, particularly beyond only C, N, and P content, is currently available.

 Cryosphere algae ionomes or elemental profiles can contribute to our understanding of these taxa’s adaptations to the nutrient conditions of terrestrial cryosphere environments. Ionomics, described as the “functional genomics of elements” by Baxter (2010)^24^, quantitively characterises the presence and abundance of ions (including macro- and micronutrients) in cells. An ionome highlights the nutritional status of a cell, thus giving insight into the cell’s functional state, with an expectation that ionomes will vary between cell types based on intrinsic factors, such as genomic adaptations (including evolutionary history), and developmental and life stages^25^. For example, the presence and quantity of nutrients in cells can be used to derive important information regarding their cellular makeup, such as enzyme activity with antioxidants (micronutrients such as Cu and Zn are important enzymatic co-factors), and photosynthesis (with Mg being essential for the stability of chlorophyll^26^). Therefore, a cell’s nutrient balance provides insight into its life history and cellular function. Furthermore, ionomes are plastic, varying based on changeable extrinsic factors, in particular environmental nutrient availability, for example with the adaptation to environments with high trace metal  concentrations^27^. A large body of work on the ionome of *Chlamydomonas reinhardtii*, a model mesophile chlorophyte algae, and a close relative of chlorophyte cryosphere algae, already exists.^2,28,29^. To identify how cryosphere algae have adapted their ionomes to cryosphere conditions, *Chlamydomonas* is therefore an excellent comparison model for non-cryosphere algae.

Traditionally, inductively coupled plasma-mass or optical emission spectrometry (ICP-MS or ICP-OES) methods are used to quantify ionomes in bulk samples. However, recently, single-cell (sc) ICP-MS has become a powerful and fast tool for elemental and nanoparticle analysis in individual cells, allowing for the identification and elucidation of biological phenomena at a single cell level^30–36^. Indeed, relevant insights into the distribution of endogenous  and exogenous elemental contents in cell populations, as well as the uptake of metallodrugs and nanoparticles, have revealed important cell-to-cell variance^37–43^. If a time-of-flight mass spectrometer is used, the approach is referred to as single-cell inductively coupled plasma time-of-flight-mass spectrometry (sc-ICP-ToF-MS). The use of a time-of-flight analyser offers unparalleled time-resolution performance, as it allows the *quasi*-simultaneous  detection of multiple elements in one single cell,  thus enabling investigations of single-cell ionomics^44–46^. Von der Au et al. (2020)^46^, evaluated the single-cell ionomes of the freshwater diatom alga *Cyclotella meneghiniana* via sc-ICP-ToF-MS and provided a deeper insight into its metal composition and response to metal stress exposure. These results, combined with multivariate data analysis, allowed the differentiation of three diatom species as well as cell distinction based on metal exposure^46^. Furthermore, a recent  study on how P is accumulated by a single-celled yeast—*Saccharomyces cerevisiae* - and how its uptake and distribution may be influenced by heavy metals also used  sc-ICP-ToF-MS^45^.

Altogether, these studies show that sc-ICP-ToF-MS can provide new prospects towards single-cell ionomes, helping us  understand cellular responses to environmental nutrient stress, and allowing the possibility to determine cell species based on the patterns of elemental content in the cells. However, so far, a comparison between different algal cell types is lacking. To address this knowledge gap, in this study, we present the ionomes (P, Mg, Ca, Cu, Fe, Mn and Zn) of three chlorophyte algae species isolated from the terrestrial cryosphere (*Microglena sp*., *Raphidonema sempervirens*, and *Deuterostichococcus sp*.) and compare them to the ionomes of two mesophile chlorophyte algae (*Acutodesmus obliquus* and *Chlamydomonas reinhardtii*) using sc-ICP-ToF-MS. We establish the first ionomes of cryosphere algae, including the quantification of intracellular trace metals and address the effect of P-starvation on the elemental contents and distributions for two of the cryosphere algal species.

## Materials and methods

### Reagents and solutions

Ultrapure water (Milli-Q water, resistivity 18.2 MΩ.cm) used for the preparation of all solutions came from a Milli-Q Advantage A10 System (Merck, Darmstadt, Germany). Pro-analysis grade HNO_3_ (65–68 % (*v/v*)) and HCl ( ≈  30 % (*v/v*)) (Chemsolute®, Th. Geyer (Berlin, Germany)) were further purified by double sub-boiling distillation.

The reagents NaNO_3_, MgSO_4_.7H_2_O, NaCl, K_2_HPO_4_, KH_2_PO_4_, CaCl_2_.2H_2_O, ZnSO_4_.7H_2_O, MnCl_2_.4H_2_O, MoO_3_, (NH_4_)_6_MoO_3_, CuSO_4_.5H_2_O, Co(NO_3_)_2_.6H_2_O, CoCl_2_.6H_2_O, H_3_BO_3_, Na_2_EDTA.2H_2_O (Titriplex III), KOH, FeSO_4_.7H_2_O, H_2_SO_4_, H_2_NC(CH_2_OH)_3_, Tris(hydroxymethyl)-aminomethan, NH_4_Cl, CH_3_COOH used for preparing the 3N-BBM (Bold’s Basal Medium) and TAP (Tris-Acetate-Phosphate) algal culture media were obtained from Merck (Darmstadt, Germany) or Thermo Fisher Scientific (Waltham, USA).

We used CertiPur® 1000 mg L^−1^ stock solutions of Au, Ca, Cu, Fe, Mg, Mn, P, and Zn from Merck (Darmstadt, Germany). Aliquots from the stock solutions were diluted with Milli-Q water to prepare all working standard solutions daily. A uniform, monodisperse, and colloidally stable standard suspension of gold nanospheres (AuNPs) with a size of 60.0 ± 4.0 nm in aqueous 2 mM sodium citrate solution at a concentration of 0.05 mg mL^−1^ or 0.254 mmol L^−1^ was purchased from nanoComposix (San Diego, USA). An Allpax ultrasonic bath (Papenburg, Germany) was used to sonicate the standard suspensions of spherical AuNPs before  diluting with Milli-Q water to prepare the working standard solutions.

Polyethylene flasks, conical bottom polypropylene tubes (Falcon), conical bottom microtubes (Eppendorf) and glassware were pre-cleaned by submerging them in 10 % (*v/v*) HNO_3_ for 24 h and rinsing multiple times with Milli-Q water. All solutions and samples were stored in pre-cleaned containers. Sample digestion was performed in a metal-free clean laboratory with ISO class 6 at the Federal Institute for Materials Research and Testing (BAM). Sample dilutions and analysis were performed in a normal laboratory environment.

### Algal culturing

Five algal cultures were used in this study: two cryosphere algal species isolated from snow in Svalbard *Microglena sp*. (CCCryo 002b-99), *Raphidonema sempervirens* (CCCryo 011a-99), one cryosphere algal species isolated from the bare ice surface of the Greenland Ice Sheet: *Deuterostichococcus sp*. GUPI-18 (gift from A. Anesio, Aarhus University^47^), and two mesophilic chlorophyte model strains *Chlamydomonas reinhardtii* (SAG 33.89) and *Acutodesmus obliquus* (SAG 276-1). All cultures were unialgal. *Microglena sp*., *Chlamydomonas reinhardtii*, and *Acutodesmus obliquus* were all axenic cultures. *Raphidonema sempervirens* (as described by CCCryo) and *Deuterostichococcus sp*. were non-axenic cultures. However, for all analysed samples, we did not find visible bacterial cells (checked by microscopy and by centrifugation). *Microglena sp. and Raphidonema sempervirens* are both psychrophilic algae (as described by CCCryo), while *Deuterostichococcus sp*. is psychrotolerant^47^. Cryosphere algae cultures were grown in a temperature- (at 4 °C to mimic the temperatures of melting snow packs and glacier surfaces) and light-controlled ( ~ 50 µmolm^-^^2^s^-1^, at 16 h/8 h light/dark conditions) incubator (Percival, USA). The light conditions were chosen to reduce any effect of high light stress on the cryosphere algal taxa (data not shown), as light stress was not an object of study. The mesophile algae were grown on a laboratory shaker (100 rpm) at room temperature ( ~ 20 °C) in continual light ( ~ 20 µmolm^-^^2^s^-1^). *Microglena*, *Raphidonema*, and *Deuterostichococcus* were cultivated in 3N-BBM (Bischoff & Bold, 1963) medium at pH 6, *Acutodesmus* in Basal medium (recipe from the Culture Collection of Algae, SAG, Georg August University of Göttingen) at pH 7.8, while *Chlamydomonas*  was cultivated in TAP medium, or TAP medium without acetic acid (TP) at pH 6^29^.

Cell handling was performed in a clean biohood (Faster, Italy) and cell separation and washing were done either with a Sigma 1-14 k (Osterode am Harz, Germany) or an Eppendorf (Wesseling-Berzdorf, Germany) centrifuge (used at room temperature for the mesophilic strains, and at 4 °C for the cryosphere algae). The cultures were harvested for sc-ICP-ToF-MS during the exponential growth phase. The analysed compositions of all used media are detailed in Supplementary Table 1. The used glassware, pipette tips, and media were sterilized in an autoclave at 121 °C for 20 min. Algal cells were observed on an Axio scope A1 (Zeiss, Germany) and an ioLight microscope (ioLight Limited, UK) and counted before sc-ICP-ToF-MS and bulk ICP-MS analysis using a hemocytometer (Thoma).

### Incubation under phosphorus starvation conditions

Two cryosphere algae, *Microglena sp*. and *Raphidonema sempervirens*, were also used in targeted P-starvation experiments. Cells were collected via centrifugation during  the exponential growth phase and thoroughly washed to remove all standard 3N-BBM medium. Cells were subsequently resuspended either in fresh P-free 3N-BBM medium (P-starved samples) or back in standard 3N-BBM medium (control samples) and incubated at 4 °C for 5 days (120 h). This experiment was undertaken twice, each time with triplicates for both species.

### Single-cell mass spectrometric analyses

For single-cell analysis, a *microFAST* single-cell autosampler from Elemental Scientific (Omaha, USA) was coupled to an *icp*TOF 2R ICP-ToF-MS instrument (Tofwerk AG, Thun, Switzerland) equipped with a 1 mm quartz injector, quartz torch, double cone interface, and a Q-cell technology for suppression of matrix ions. A specific high-sensitivity single-cell sample introduction system from Elemental Scientific (Omaha, USA) consisting of a Cytoneb nebulizer and a Cytospray chamber was used. High-purity argon (99.999 %) was used for all analyses.

For sc-ICP-ToF-MS analysis, aliquots of each cell suspension were centrifuged at 1500 × *g* for 5 min at 4 ^◦^C (cryosphere algae) and at 20 ^◦^C (mesophilic algae), the supernatants were carefully discharged, and the cell pellets were washed four times with ultrapure water to remove remnant salts from the media. Subsequently, cells were resuspended in ultrapure water, homogenized, counted using a hemocytometer, and diluted to a cell density of ~2 × 10^5^ cells mL^−1^ immediately before the sc-ICP-ToF-MS analysis. To guarantee a reliable analysis of the individual alga in each sample, each biological replicate was measured in technical triplicates (except *Microglena*). For all measurements ultrapure water was used as a blank. No cell staining or labelling was required, as single cells were detected based on their natural elemental fingerprint^46^.

To optimise sensitivities and resolutions, the ICP-ToF-MS instrument was tuned daily before measurements with an aqueous multi-element standard solution containing 1 µg L^−1^ of Ba, Bi, Ce, Co, In, Li, and U each in a matrix of 2 % (*v/v*) HNO_3_ plus 0.5 % (*v/v*) HCl (Thermo Scientific). For thorough cleaning before the measurements, 1 % (*v/v*) HNO_3_ and ultrapure water were injected (at least 3 times each) into the autosampler loop, sample injection tube, and the single-cell introduction system. The 1 % (*v/v*) HNO_3_ solution and ultrapure water were also used to clean the system between each algal sample analysis. The transport efficiency (TE) of the cells was determined daily by measuring 1:100,000 diluted suspensions of AuNPs of 60.0 ± 4.0 nm in deionized water (particle size approach) and a TE of up to 90 % was achieved. The optimized parameters for the method are given in Supplementary Table 2. The collision/reaction cell was pressurized with hydrogen gas with an optimized gas flow rate of 3.00 mL min^−1^ to remove the polyatomic interferences. Calibrations for sc-ICP-ToF-MS spectra were performed with dissolved multi-element standards prepared in deionized water containing Ca, Cu, Fe, Mg, Mn, P, and Zn in concentration ranges of 1.0–100.0 µg L^−1^. Calibration curves of dissolved Au in water at a  linear concentration range of 0.1–10.0 µg L^−1^ were also carried out to create the transformed calibration curves. Cell samples with a cell concentration of around 2 × 10^5^ cells mL^−1^ were introduced into the system at a controlled sample flow rate of 10 μL min^−1^. The sc-ICP-ToF-MS data analysis software TofPilot (Tofwerk AG, Switzerland) was used to extract the data out of the time-resolved signals and to calculate the elemental mass data of single cells.

### Cell digestion and bulk ICP-MS analyses

To optimise and make the comparison between single-cell ICP-ToF-MS and bulk ICP-MS more robust, both analyses were undertaken on the same samples to avoid any impact of varying growth conditions and handling^48^. Therefore, aliquots of the same samples that were washed and analysed by sc-ICP-ToF-MS were also digested for bulk analysis by ICP-MS on a hotplate at 120 °C for 3 h by adding a mixture of 2 mL of HNO_3_, 0.5 mL of HCl, and 0.5 mL of H_2_O_2_. The digested cells were diluted with 1 % (*v/v*) HNO_3_ and analyzed via an Element 2 sector-field ICP-MS (Thermo Scientific, Germany) using a routine quantitative method for determining P, Mg, Ca, Cu, Fe, Mn, and Zn. Samples were run in duplicate for each biological replicate along with five digestion blanks.

### Cell biovolume and carbon content calculation

Aliquots of the same samples that were analysed by sc-ICP-ToF-MS were fixed in 2 % Glutaraldehyde (*v/v*), stored at 4 °C and analysed using a FlowCam 5000 (Yokowaga Fluid Imaging Technologies). The FlowCam instrument is equipped with a 10x objective and data was analysed using the Visual Spreadsheet 5 software. The measurement and data analyses procedure followed the methods detailed in Peter et al. (2024)^49^ and Feord et al. (2025)^50^. In brief, the flowrate was set to 250 mL min^−1^, the distance to nearest neighbour set to 0.1 µm, a pixel intensity threshold for dark & light pixels set to 15, and the close hole iterations set to 5. After imaging, cells were randomised, and the widths and lengths of ~900 cells per sample were measured using imageJ^51^. Cell biovolumes (in µm^3^) in each sample were subsequently calculated using the following equations^52^: for spheroid cells (*Acutodesmus obliquus, Chlamyomonas reinhardtii, Microglena* sp. and *Deuterostichococcus* sp.):and for cylindrical cells (*Raphidonema sempervirens*):

Using the so evaluated biovolumes, the individual cell carbon contents (in pg) were calculated using the following equation^53^:

$${Carbon}\,=\,0.109* {biovolum}{e}^{0.991}$$

### Multivariate analysis data

The  seven fingerprint elements (P, Mg, Ca, Cu, Fe, Mn and Zn) for the five algal species were considered for canonical discriminant analysis. The single-cell elemental masses for the biological triplicates of each algal species were merged resulting in a multi-elemental mass dataset with dimensions of 132,554 × 7. Missing values were replaced with LOD values for each element and the data matrix was log-transformed. Then, the linear discriminant analysis was performed in MATLAB software following Ballabio and Consonni (2013)^54^, and the canonical variable score and loading values were obtained.

## Results

### Single-cell signals and fingerprint elements

**Fig. 1:**
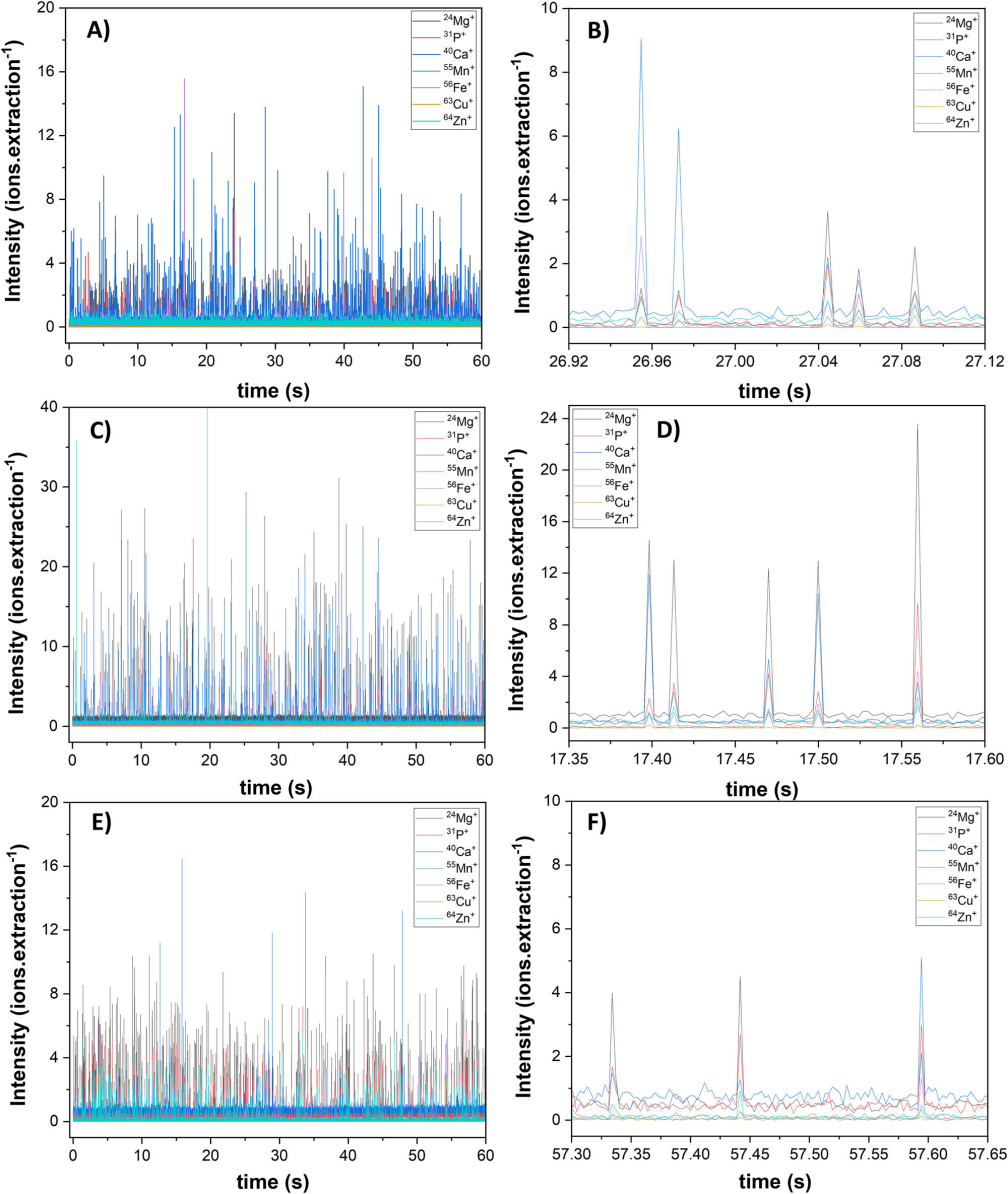
Simultaneous time-resolved transient signals of the fingerprint elements Mg, P, Ca, Cu, Fe, Mn, and Zn corresponding to cell events of the algal species. *Raphidonema sempervirens* (**A**, **B**), *Microglena* sp. (**C**, **D)** and *Deuterostichococcus* sp. (**E**, **F**). **A**, **C** and **E** show the combined signals acquired over 60 s (part of the evaluated measurement time), while **B**, **D**, **F** show zoomed-in sections of the transient signals showing the presence of the target elements.

The time-resolved transient signals from the sc-ICP-ToF-MS analysis of the three cryosphere algae - *Raphidonema sempervirens* (Fig. 1A)*, Microglena* sp. (Fig. 1C) and *Deuterostichococcus* sp. (Fig. 1E) are presented as simultaneous signals for seven intrinsic elements - P, Mg, Ca, Cu, Fe, Mn and Zn. These elements were identified as fingerprint elements due to their essential cell functions. Each cell produced multi-elemental peaks at the same integration time, as can be better observed in the zoomed-in sections of the transient signals for *Raphidonema* (Fig. 1B), *Microglena* (Fig. 1D) and *Deuterostichococcus* (Fig. 1F) - with intensities proportional to their absolute amounts. We assigned an event to an alga when three of these elements were detected simultaneously (P, Mg, and Mn). The efficient removal of the culture medium from the algae cell samples through washing was demonstrated by the low background baselines for the seven fingerprint elements (Fig. 1). Transient signals for Potassium were sometimes observed, with most of these events not appearing simultaneously with the other element transient signals. We therefore classified these as random spikes and excluded them from any further interpretations.

The accurate quantification of multi-elements within single cells required the optimization of important instrumental parameters. The spray chamber used in this work has a makeup gas inlet positioned to create a tangential flow to the spray chamber walls, thus preventing cells from colliding with and sticking to the walls. To ensure that each simultaneous multi-elemental transient signal corresponded to one individual algal cell, the minimum integration time (3 ms) available in the instrument was selected, which was longer than the peak duration for one cell in ICP-MS (0.2–0.5 ms)^55^. Although longer times can increase the probability of detecting multiple events and increase difficulties in distinguishing cell signals from background signals, the analyses of two cells simultaneously was avoided by keeping the density of the cells in the samples low ( ~ 2 × 10^5^ cells mL^−1^) and controlling the sample flow rate (10 μL min^−1^), leading to a reaching cell rate introduced into the sc-ICP-ToF-MS of approximately 2000 cells min^−1^. This corresponds to less than 1 cell per 10 ms, which is > 3 times longer than the used 3 ms integration time. To avoid algae cells aggregating and settling down in the sample vials, sample resuspension/mixing was carried out automatically by the autosampler probe before the sample injection. In addition, in terms of morphological integrity, the centrifuge g-force used for cell separation during the washing process was suitable to avoid cell rupture (1500 x g) and, after passing through the nebulizer (nebulizer gas flow rates up to 0.30 L min^−1^) the individual cells were still preserved as confirmed by  post-analysis optical microscopy. Analytical calibration curves for the evaluated isotopes of the elements showed good linear correlation coefficients (R^2^ >  0.99). The transport efficiency (TE), calculated by the particle size approach, reached up to 90 %. The limits of detection (LOD) ranged from 0.04 fg cell^–1^ (Cu) to 2.60 fg cell^–1^ (P) and the limit of quantification (LOQ) ranged from 0.14 fg cell^–1^ (Cu) to 8.68 fg cell^–1^ (P) (Supplementary Table 3).

### Discriminant analysis and multi-elemental mass distributions in single cells of mesophilic algae and algae isolated from the terrestrial cryosphere

**Fig. 2:**
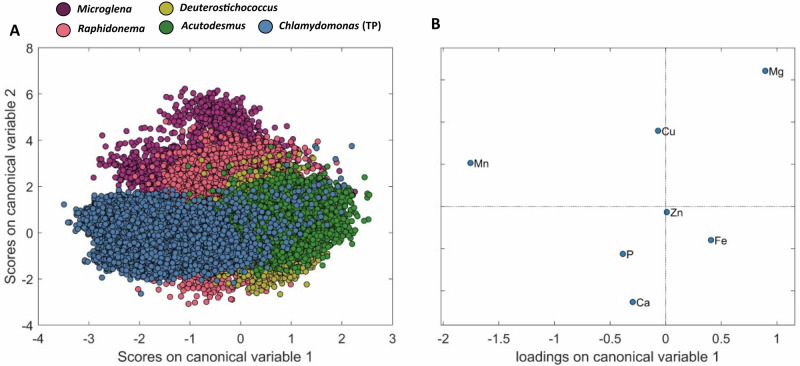
Discriminant analysis applied for the five different algal species investigated - cryosphere algal species - *Raphidonema sempervirens*, *Microglena* sp. and *Deuterostichococcus* sp. - plus mesophilic algae - *Chlamydomonas reinhardtii* and *Acutodesmus obliquus*. Canonical score plot of the discriminant analysis **A** and loadings **B**.

The masses of the seven fingerprint elements (Mg, P, Ca, Cu, Fe, Mn, and Zn) were successfully determined in all algal species by sc-ICP-ToF-MS, with the number of detected cell events varying between the species and ranging from 379 to 7715. We tested the feasibility of separating our five different algal species using a  multivariate analysis, based on the defined seven fingerprint elements. To do this, we merged the multi-elemental mass datasets of the five algal species (three cryosphere and two mesophile algal species), log-transformed them (replacing missing values with LOD values for each element), applied a discriminant analysis and plotted them as a canonical score plot (Fig. 2). We found incomplete separation among the five species (Fig. 2A), indicating differences between the ionomes of the species which were not large enough to fully distinguish them. Generally, our data do not give an indication of individual determination of environmental grouping (the cryosphere algae did not create a separate group). Moreover, the two mesophilic strains clearly separated in the discriminant analysis (Fig. 2A). We identified Mg and Mn levels as the factors with the greatest effect on the discrimination of algal species (Fig. 2B).

**Fig. 3:**
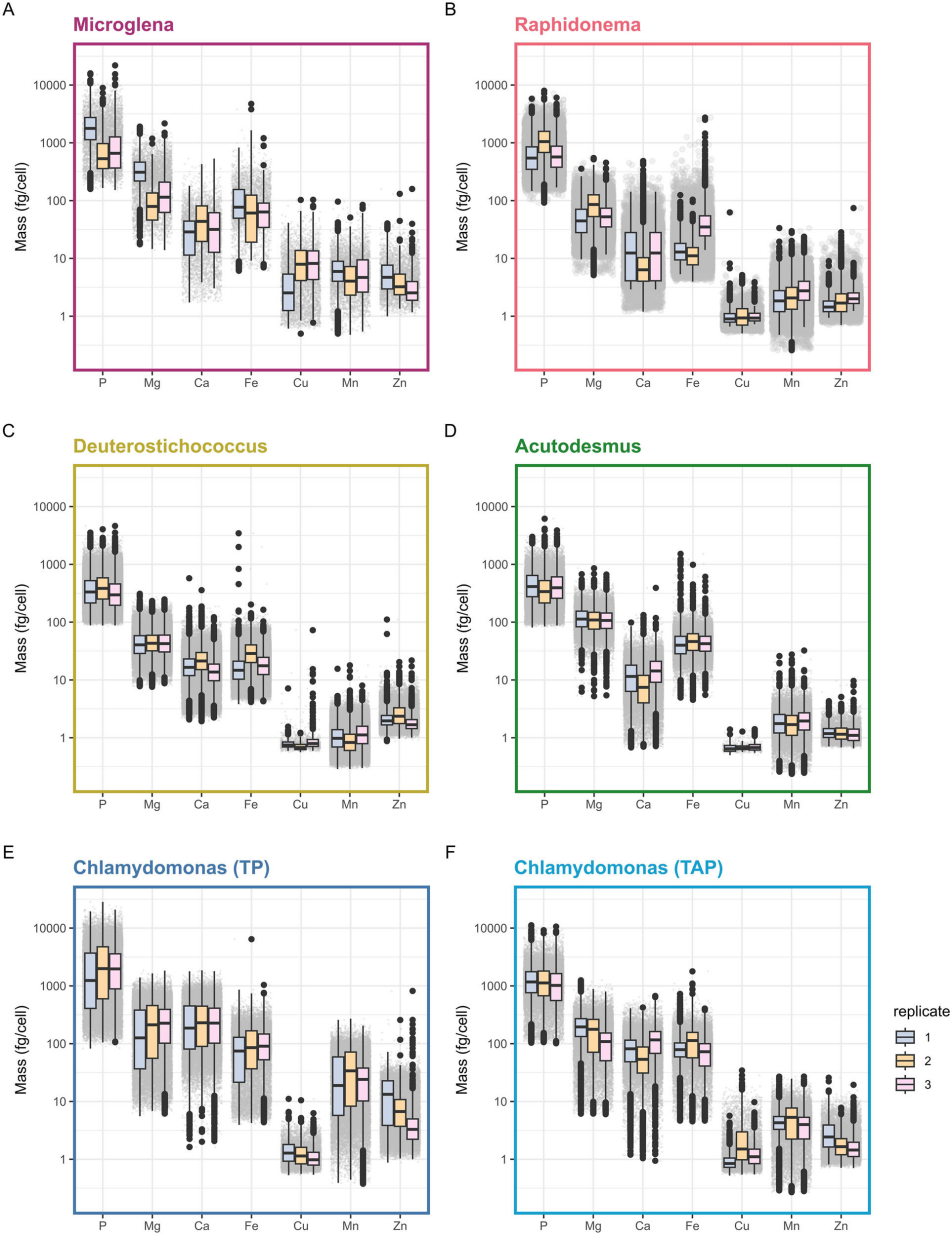
Multi-elemental contents (P, Mg, Ca, Cu, Fe, Mn and Zn) in single cells of the algal species analysed by sc-ICP-ToF-MS in fg cell^−1^. Biological replicates are plotted with separate boxplots. Cryosphere algae are **A** *Microglena* sp. (purple), **B** *Raphidonema sempervirens* (pink), **C** *Deuterostichococcus* sp. (yellow), and mesophilic species are **D** *Acutodesmus obliquus* (green) *and Chlamydomonas reinhardtii* grown in **E** TP (dark blue) and **F** TAP media (light blue).

Among the cryosphere species, *Microglena* presented the highest mean P, Mg, and Ca contents, 1365.52, 213.14, and 46.48 fg cell^−1^, respectively (Fig. 3A). This feature of *Microglena* promoted its slight separation from the other algal species in the discriminant plot (Fig. 2A). Considering all the biological replicates, P, Mg, and Ca mean contents for *Microglena* were at least 1.5 times higher than those found for the other two cryosphere species, *Raphidonema* (867.53, 68.96 and 17.12 fg cell^-1^) and *Deuterostichococcus* (413.45, 48.30, and 19.35 fg cell^−1^, Fig. 3B-C). However, the mean content of P for *Microglena* was around two times lower than that in the mesophile *Chlamydomonas* cultivated in TP medium (2780.29 fg cell^−1^) but similar to *Chlamydomonas* cultivated in TAP medium (1326.26 fg cell^−1^; Fig. 3E-F). Mg mean contents were comparable among *Microglena* (213.14 fg cell^−1^) and *Chlamydomonas* cultured both in TP and TAP media (267.23 and 171.19 fg cell^−1^). In contrast, the Ca mean content was lower for *Microglena* compared to *Chlamydomonas* in TP and TAP media (295.43 and 92.27 fg cell^-1^). *Acutodesmus* cells had lower P, Mg, and Ca (466.96, 120.10, 7.78 fg cell^−1^) contents compared to *Microglena* and *Chlamydomonas*. The co-location of *Deuterostichococcus and Raphidonema* in the discriminant plot (Fig. 2A) is likely associated with the comparable mean contents of P, Mg and Ca of these two cryosphere species (Fig. 3B-C).

**Fig. 4:**
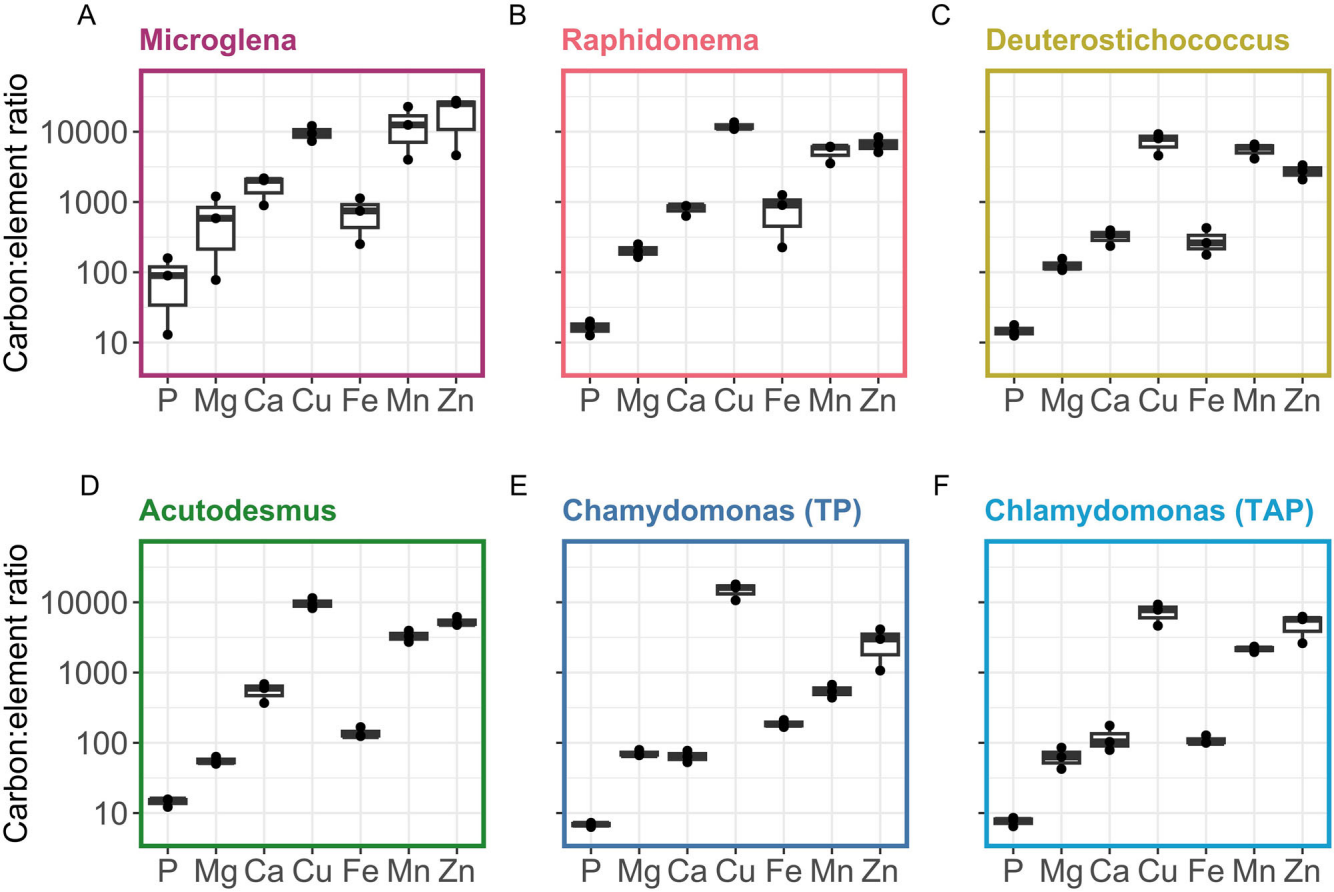
Boxplots for the calculated Carbon: element mass ratios in single cells of all algal species. Each boxplot presents data for the biological triplicates. Cryosphere algae are **A** *Microglena* sp. (purple), **B** *Raphidonema sempervirens* (pink), **C** *Deuterostichococcus* sp. (yellow), and mesophilic species are **D** *Acutodesmus obliquus* (green) and *Chlamydomonas reinhardtii* grown in **E** TP (dark blue) and **F** TAP media (light blue).

*Microglena*’s high P, Mg, and Ca contents are likely a product of a significantly higher biovolume and carbon content compared to the other species (Fig. 4, Supplementary Fig. 1, ANOVA, followed by a post-hoc Tukey *p* < 0.05). However, *Chlamydomonas* cultures still accumulated larger quantities of P, Mg and Ca despite their smaller size compared to *Microglena* (Supplementary Fig. 1), especially for cells cultured in TP medium (Fig. 3E-F). When calculating carbon to mean elemental mass ratios for each species, we found that the cryosphere algae generally had a higher calculated C:P ratio compared to the mesophilic *Chlamydomonas* (but not *Acutodesmus*). In fact, *Microglena* had a significantly higher C:P compared to all other species (ANOVA, followed by a post hoc Tukey test, *p* < 0.05; Fig. 4). The three cryopshere snow algal species all had C:Mg ratios that were higher ( > 100) than those for mesophilic algae ( < 100), and significantly higher for *Microglena* (ANOVA, followed by a post hoc Tukey test, *p* < 0.05; Fig. 4). In addition, C:Ca ratios were an order of magnitude higher in *Raphidonema* and *Microglena* (~1000) compared to both *Chlamydomonas* cultured in TAP and TP media ( ~ 100, Fig. 4). These datasets suggest that the cryosphere algal species generally contain lower concentrations of P, Mg and Ca compared to the mesophilic species. The relative difference in each species’ P, Mg and Ca content is also reflected in the media they were grown in (Supplementary table 1).

**Fig. 5:**
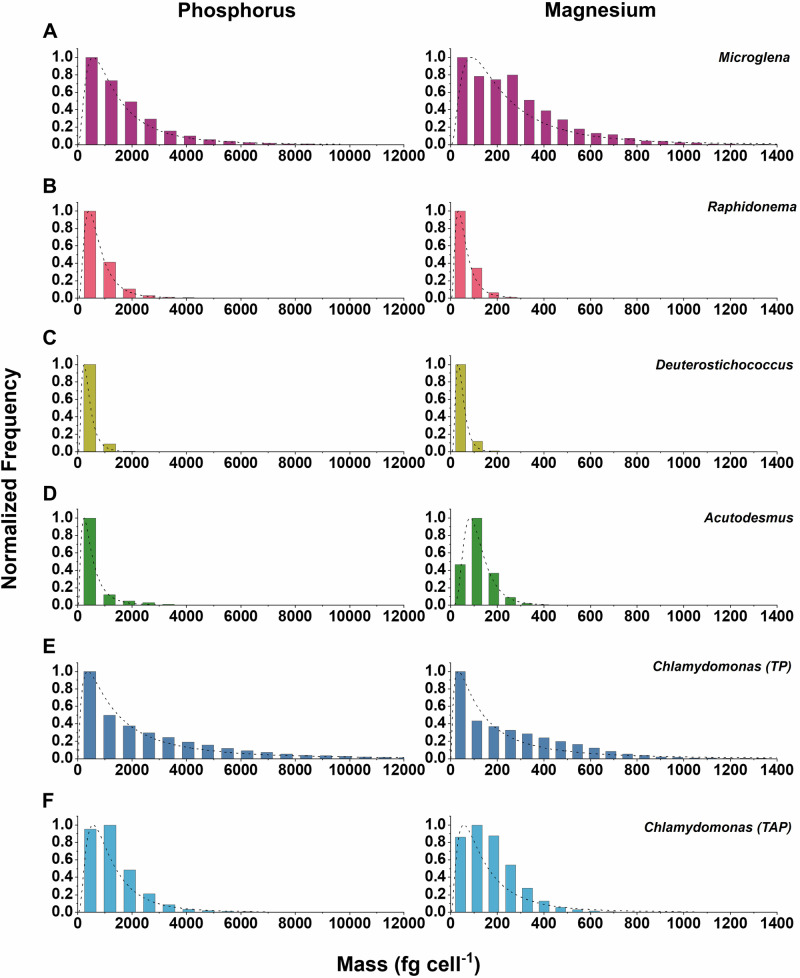
Normalized frequency mass distributions of P and Mg in cryosphere and mesophile algal cells of selected replicates. **A** *Microglena* sp., **B** *Raphidonema sempervirens*, **C** *Deuterostichococcus* sp., **D** *Acutodesmus obliquus*, **E** *Chlamydomonas reinhardtii* (TP), **F** *Chlamydomonas reinhardtii* (TAP). Each distribution was fitted by a log-normal function (dashed lines).

Regarding elemental mass distributions, mass ranges of all elements followed a log-normal distribution, which can be observed in the P and Mg histograms (Fig. 5). Among the snow algae, *Microglena* presented the widest distributions of P (366.94 to 1128.81 fg cell^−1^), Mg (60.90 to 220.27 fg cell^−1^) and Ca (10.74 to 63.31 fg cell^-1^) masses, exhibiting the high heterogeneity of its cell population as also visible in the discriminant analysis and coefficients of variation (25–75 percentile for one of the replicates, Supplementary Tables 4–6; Figs. 2A, 3A, 5A, Supplementary Fig. 2A-B). However, even broader mass distribution ranges for P (503.08 to 4516.60 fg cell^−1^), Mg (46.29 to 454.46 fg cell^-1^) and Ca (100.36 to 463.89 fg cell^−1^) were observed for the mesophilic *Chlamydomonas* grown in TP medium (25–75 percentile for one of the replicates, Supplementary Tables 4–6; Figs. 3E, 5E Supplementary Fig. 2A-B). However, we did not identify a correlation between sample elemental mass variation and sample biovolume mass variation across all species and replicates (Supplementary Fig. 2B). Therefore, algal cultures with higher biovolume variability did not equate to higher elemental variability.

Regarding micronutrients, the mean mass of Cu in single cells of *Microglena* (8.30 fg cell^−1^) was significantly higher than those found for all other algal species (at least five times more, ANOVA, followed by a post hoc Tukey, *p* < 0.05), which is also visible in the discriminant analysis loadings (Fig. 2B, Fig. 3A and Supplementary Table 7). Cu contents for single cells of *Microglena* varied from 4.01 to 12.83 fg cell^−1^ (25–75 percentile for one of the replicates–Supplementary Table 7, Fig. 3A). However, all algal species exhibited a similar C:Cu ratio ( ~ 10^4^; Fig. 4), suggesting they have similar concentrations of this element.

Similar to Cu, the mean Fe content was the highest for *Microglena* sp. (124.33 fg cell^-1^). Fe in *Microglena* sp. was around four times higher than the contents  found for *Raphidonema* (26.21 fg cell^−1^) and *Deuterostichococcus* (23.13 fg cell^−1^), yet only ~ 20 % higher than those found for *Chlamydomonas* in TP and TAP media, 101.74 and 93.78 fg cell^−1^, respectively (Supplementary Table 8 and Fig. 3A-C, E-F). However, calculated C:Fe ratios were between 100 and 1000, with the following trend: *Microglena = Raphidonema > Deuterostichococcus > Acutodesmus > Chlamydomonas* (TP) > *Chlamydomonas* (TAP).

 Mean single-cell Mn content in *Microglena* sp. (6.87 fg cell^-1^, varying between 3.82–8.57 fg cell^−1^, 25–75 percentile for one of the replicates) was at least two times higher than for other cryosphere algal species, *Raphidonema* (2.69 fg cell^−1^) and *Deuterostichococcus* (1.11 fg cell^−1^), but with a higher C:Mn ratio (Figs. 3A-C, 4, Supplementary Table 9). However, Mn content in *Chlamydomonas* cells grown in TP medium (35.52 fg cell^−1^) was almost one order of magnitude higher than for the same species grown in TAP and was significantly higher than all other cultures including *Microglena* (ANOVA, followed by a post hoc Tukey, *p* < 0.05), also visible in the discriminant analysis loadings (Figs. 2B, 3E-F, Supplementary Table 9).

The mean content for Zn (4.90 fg cell^−1^) in *Microglena* (Supplementary Table 10, Fig. 3A) was more than two times higher than those found for other cryosphere algal species (2.08 and 2.27 fg cell^-1^) and *Chlamydomonas* (2.21 fg cell^−1^) in TAP medium. *Chlamydomonas* cells in TP medium contained the highest Zn concentrations, which were significantly higher than all cultures except *Microglena* (ANOVA, followed by a post hoc Tukey, *p* < 0.05). Conversely, *Microglena* had a significantly higher C:Zn ratio  compared to all other algae except *Raphidonema* (Fig. 4, ANOVA, followed by a post hoc Tukey, *p* < 0.05).

### Single-cell ICP-ToF-MS *versus* bulk analysis by ICP-MS

**Fig. 6:**
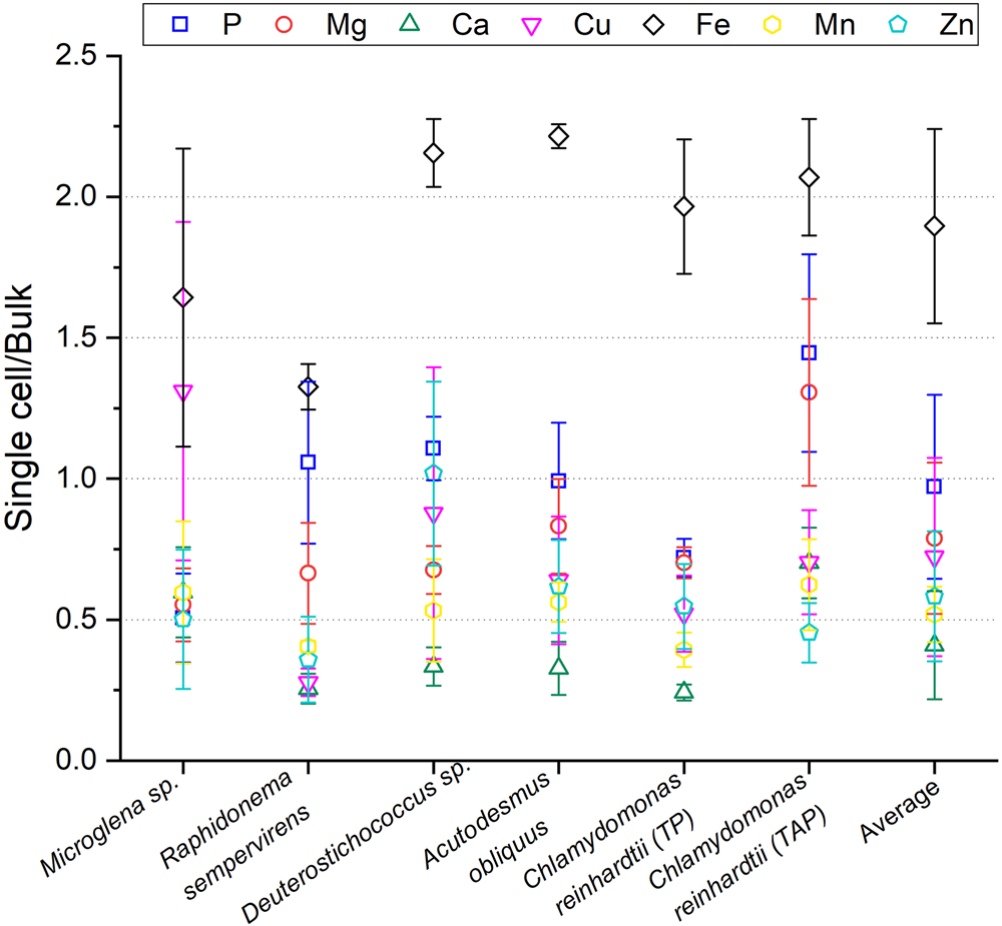
Ratios between the mean masses obtained by single-cell ICP-ToF-MS to those from bulk analyses by ICP-MS for P, Mg, Ca, Cu, Fe, Mn, and Zn in all algal samples.

The accuracy of the single-cell ICP-ToF-MS results was tested against bulk analysis of acid digested cells analysed by ICP-MS. The ratios between the mean masses found by sc-ICP-ToF-MS to those found by bulk ICP-MS in all algal samples ranged from 0.24 for Ca in *Chlamydomonas* to 2.22 for Fe in *Acutodesmus* (Fig. 6). The average value of all ratios was 0.84, suggesting around ~20 % lower values quantified by sc-ICP-ToF-MS compared to bulk ICP-MS.

### Effect of Phosphorus starvation on the *Microglena* sp. and *Raphidonema sempervirens* ionomes

**Fig. 7:**
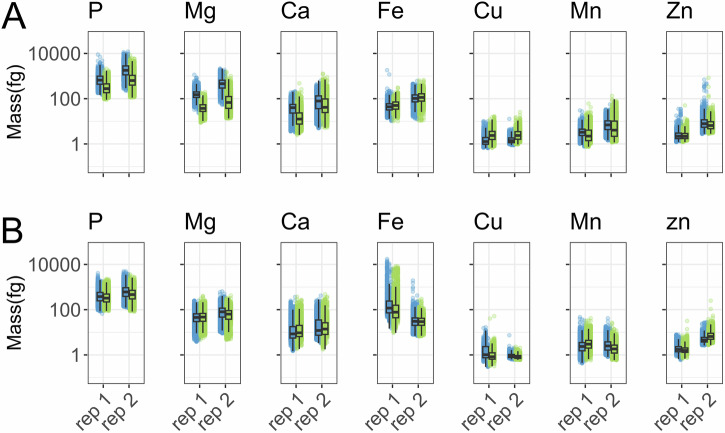
Distribution and contents of P, Mg, Ca, Fe, Cu, Mn, Zn (in fg) in single cells of two cryosphere algal species under P-replete conditions (blue) and P-starved conditions (green). **A** *Microglena sp*. and **B** *Raphidonema sempervirens.* For each case, both biological replicates (1,2) are plotted. Abbreviations: rep = biological replicate.

We assessed the robustness of the sc-ICP-ToF-MS as a method for quantifying single-cell ionomes within biological experiments, by analysing *Microglena* and *Raphidonema* cells starved of P for five days (Fig. 7). This way, we evaluated if sc-ICP-ToF-MS can be an optimal method to differentiate the nutrition status of cells from the same species that were treated differently.

We used two statistical approaches to assess the effect of P-starvation treatments for each species: a) performing a PERMANOVA for each element analysed (pooling all events from all technical triplicates for both biological replicates) and b) calculating the average of each element for each technical replicate and undertaking an unpaired student’s t-test for each element. For both species, the PERMANOVA analysis, taking full advantage of the heterogeneous data provided with the use of sc-ICP-ToF-MS analysis, indicated more variation between treatments than unpaired t-tests (Supplementary Table 11). However, for *Raphidonema*, there was little consistency in trends for pairwise comparisons across both biological replicates despite low *p*-values from the PERMANOVA analysis (Supplementary Table 12).

**Fig. 8:**
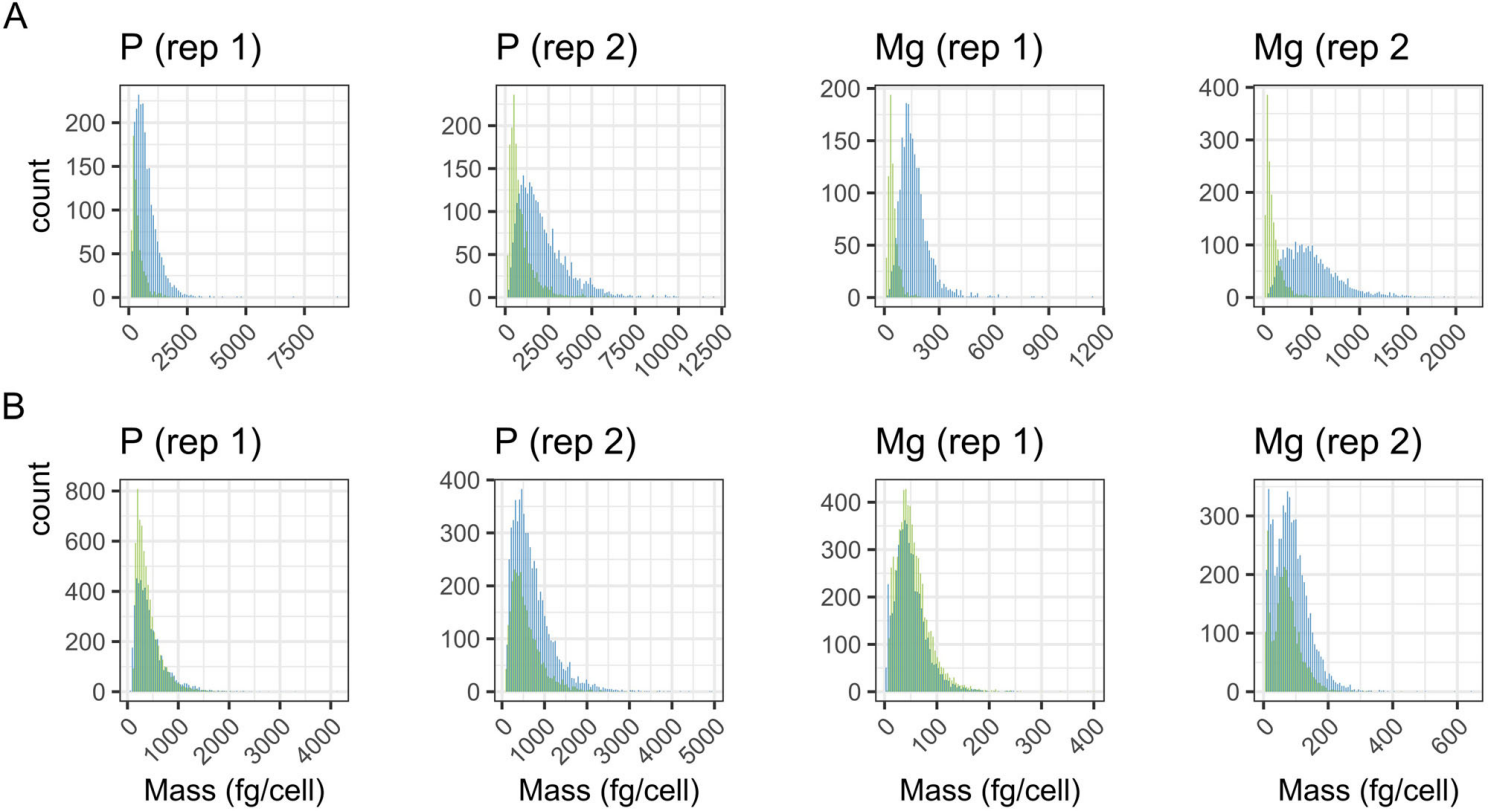
Mass distribution histograms of P and Mg in **A** *Microglena sp*. and **B** *Raphidonema sempervirens* in P-replete (blue) and P-starved conditions (green). Abbreviations: rep = biological replicate.

Basing our results on the more conservative unpaired t-tests results that were consistent across both biological replicates, we found a significant reduction of P ( > 50 %), Mg ( > 70 %), and a significant increase in cellular Cu ( > 75 %) for *Microglena* when P-starved (Figs. 7A, 8A). Moreover, the mass distribution ranges for P and Mg in *Microglena* were considerably narrower in P-starved than in P-replete conditions, as can be observed in the mass histograms (Fig. 8A). In contrast, we found no consistent significant trends for *Raphidonema* for pairwise comparisons (Fig. 7B), and the mass distribution ranges for P and Mg were similar (Fig. 8B). For both taxa, it is unlikely that P-starvation affected  cell size, as such a response would also impact other elements (Fig. 7). Globally our results indicate an ionome response for *Microglena* under P-starvation and a general confirmation that we can detect intra-species changes in ionomes using sc-ICP-ToF-MS.

## Discussion

We quantified, for the first time, the ionomes (or elemental profiles) of algae isolated from the terrestrial cryosphere, providing essential insight into the cellular homeostasis of key biological ions. Applying sc-ICP-ToF-MS, we highlighted the importance of using a single-cell approach, as it offers a more in-depth view of the cell population compared to bulk analyses from total digestions; our data also revealed the natural cellular heterogeneity in cultures, providing increased biological resolution over conventional ICP-MS.

We validated our sc-ICP-ToF-MS workflow by comparing single-cell masses to total bulk digestions. Although we observed divergences between ratios for sc-ICP-ToF-MS and bulk analysis by ICP-MS (between 0.24 and 2.22; Fig. 6), the overall magnitudes were similar to other such comparisons reported in the literature for yeasts, epithelial and cancer cells: 0.45 to 1.51 (mean = 0.81), 0.61 to 1.36 (mean = 0.98), 0.59 to 2.28 (mean = 1.00), and 0.23 to 1.23 (mean = 0.82)^45,56–58^. One explanation for the difference between single-cell and bulk analysis could be a technical difference between the obtained transport efficiency using Au nanoparticles and the true transport efficiency achieved for the cells. A biological explanation could be the changes in the condition of the cell suspension over time, since the ions may be excreted from the cells due to osmotic imbalance (bulk analysis after digestion cannot differentiate dissolved extracellular ion and  ion in the cell)^39,45,57^. In the current study, we measured cells with sc-ICP-ToF-MS as rapidly as possible after washes ( ~ 20 minutes), which is recommended to reduce cellular ion loss.

Our single-cell data presents important evidence regarding the cell-to-cell variation in elemental masses and biovolume in algal cultures from the terrestrial cryosphere and algal monocultures generally. The log-normal distribution of all elements and for the studied taxa (Fig. 5) concurs with the knowledge that living cells mostly follow a log-normal size/growth distribution, indicative of a culture made up of cells at different growth stages^59,60^. Therefore, the cell-to-cell variation in elemental masses (Figs. 2, 3, 5) can likely be linked to variations in cell sizes and nutritional status associated with cell cycle stage and cell age, in particular for cell cultures that are not synchronous. Furthermore, for algae of the *Chlamydomonales* order (*Chlamydomonas* and *Microglena*), there is also the influence of life cycle stage which affects cell size, morphology, and nutrient uptake and use^61–64^. Indeed, we found the highest coefficient of variation for biovolume in the *Chlamydomonales* species compared to the other taxa tested (Supplementary Fig. 2B), confirming the important contribution of life cycle stage to cell size variation. However, as higher biovolume variation did not necessarily imply higher elemental mass variation (Supplementary Fig. 2A), it is likely that morphological variation due to cell life stage is not a strong underlying cause for cell-to-cell variation in the elemental profiles.

The sc-ICP-ToF-MS and bulk ICP-MS quantified elements for the different species (Figs. 3, 6) followed the predicted trend for these elements in green algae (P > Mg > Ca > Fe > Mn/Cu/Zn), with the exception of Fe^26,65^. In particular, quantified elements, except Cu, were higher in *Chlamydomonas* cells grown phototrophically, which is fully consistent with the trend previously outlined by Esteves et al. (2023; using digestion and ICP-AES)^66^. Similarly, in a previous study with *Chlamydomonas reinhardtii* cultured in a sodium acetate-based medium, Tanaka et al., (2000)^40^ reported similar mean contents of P (2044 fg cell^−1^), Mg (293 fg cell^−1^) and Zn (3.9 fg cell^−1^), but not Fe (14.5 fg cell^-1^). However, as Ca, Mn and Cu were not quantified by Tanaka et al. (2000)^40^, it was impossible to fully compare ionomes. Therefore, in addition to the quantification of cryosphere algae ionomes, our data provides essential new insights into the cell-to-cell variability within the ionome of the model alga *Chlamydomonas* both grown heterotrophically (TAP) and phototrophically (TP). However, strikingly, the high sc-ICP-ToF-MS quantification of Fe ( ~ 100 fg/cell, ~1 × 10^9^ atoms/cell, Fig. 3E-F, Supplementary Fig. 3) was up to an order of magnitude higher than previously published data for *Chlamydomonas* grown mixotrophically, indicating a possible technical error rather than biological variation^26,27,40,65,67,68^.

The single-cell ionomes of the three cryosphere and two mesophilic algae did not cleanly separate based on habitats (Fig. 2), indicating that, at least in nutrient-replete laboratory cultures, chlorophyte algae originating from the terrestrial cryosphere did not have a significantly different ionome compared to their mesophile cousins (Fig. 3). For both algal categories presented here, it is therefore possible that the difference in taxonomy (and the associated evolutionary history) supersedes habitat conditions in terms of their influence on ionomes under nutrient-replete laboratory culture conditions. Indeed, under such conditions, different evolutionary histories (for example, belonging to the Trebouxiophyceae *vs.* the Chlorophyceae class) as well as species-specific differences, could influence cellular ionomes more than the habitat they came from.

However, the growth temperature used for the cryosphere algae compared to the mesophilic taxa likely impacted the ionomes of the studied taxa. Indeed C:P, C:Mg, and C:Ca ratios indicated that the cryosphere algae generally had lower intracellular P, Mg, and Ca concentrations. P, Mg, and Ca exist in cells in relatively high abundance to maintain a variety of cellular metabolic pathways, such as energy transfer, nucleic acid synthesis, photosynthesis and signalling^69–72^. Life in the cold will induce lower metabolic rates^73^, which could underpin a lowered cellular requirement for P, Mg and Ca. Trace metal-wise, we were not able to detect an important trend other than a high calculated C:Zn ratio in *Microglena* and *Raphidonema*, suggesting that this element may not be as biologically important for these taxa compared to the other cells analysed. Our data indicate that, except for a potential role for Zn in *Microglena* and *Raphidonema*, trace metal cellular content (and potential associated cellular trace metal use), does not appear to be impacted by temperature. It is not clear why cellular trace metals, which are essential co-factors in cellular metabolism, are not impacted by temperature, despite temperature slowing metabolism. Further work combining ionomics with other omics work, such as genomics and proteomics, is necessary to fully assess trace metal homeostasis in algae isolated from the terrestrial cryosphere.

In the future, sc-ICP-ToF-MS should be used for environmental samples, such as carotenoid-rich red snow algae, or for cultures of algae that are more environmentally abundant, for example *Ancylonema* spp. However, an important limiting factor for environmental samples is the large amount of minerals and other impurities in natural samples, as these  would interfere with measurements^6^. Furthermore, a limitation with the use of *Ancylonema* spp. cultures is that they are not axenic, as they have yeast contamination, whose cells may not be distinguishable by ICP-ToF-MS^74^.

P-starvation in *Microglena* led to reduced cellular P and Mg and increased Cu. However, we found  no reproducible (across biological replicates and statistical analysis types) important elemental changes for *Raphidonema* (Figs. 7–8). The difference in response to P-starvation of these two algal species highlights a differential adaptation to low P environments, with our *Microglena* single-cell data suggesting that *Microglena* has the ability to store both larger and more variable quantities of cellular P in contrast to *Raphidonema* (Figs. 7–8). For both P and Mg, the histogram narrowing after P-starvation indicates both a general lowering of cellular concentrations, but also a reduction in cell-to-cell variability for *Microglena* compared to *Raphidonema*. This result confirms that this method can be robustly used for experimental cultures and provides a very good example of the power of using sc-ICP-ToF-MS to study nutrient storage and homeostasis in cell cultures. Such observations have been previously reported for other algal species, including chlorophytes and diatoms^37,46^, and yeast^45^. Not only are we able to assess changes in cellular elemental content, but the high resolution of this analysis type compared to bulk digestion methods, highlights the important changes in mass distribution (Fig. 8), which are not quantifiable with bulk digestion.

P-reduction during P-starvation has been reported for many algal species, such as *Chlorella vulgaris*^75^, *Phaeodactylum tricornutum*^76^ and *C. reinhardtii*^77^. The P-starvation response identified in *Microglena* correlates with a putative hydrolysis of polyphosphates: the correlative losses of P, Mg, and Ca under P-starvation conditions (Fig. 7A) could be because Mg and Ca are known to be important polyphosphate-chelated ions^78^. The increase in Cu (Fig. 7A) could also be associated with the loss of polyphosphates, as decreases in cellular P and polyphosphates are known to also decrease the tolerance to trace metals in different algal cell types^78,79^. Furthermore, Cu can also be directly linked to the putative active hydrolysis of polyphosphates, if Cu is an important cofactor for relevant hydrolases^79^. Finally, the relationship between P, Mg, and Cu is also visible in our non-P starvation experimental samples (Fig. 3A): the comparative trends between biological replicates indicate that a lowering of P and Mg is associated with an increase in Cu.

P concentrations in terrestrial cryosphere environments are known to be low and/or limiting for biota, including algae^6,8,16,18^. A recent single-cell study of the glacier ice alga *Ancylonema*, which blooms on ice surfaces, quantified strikingly high C:P ratios highlighting the presence of both cellular P-storage molecules, as well as relatively low cellular P requirements^18^. *Microglena* may have a similar strategy. Combined with our data from various other biological replicates, which show a relatively large cell-to-cell and culture-to-culture variation (Figs. 3–5), we can hypothesise that this alga may have an important elemental homeostasis plasticity. Such a plasticity could combine both an ability to store large amounts of P and Mg, as well as metabolic adaptations which may overall reduce cellular P and Mg demands. Such a strategy could ultimately lower continual cellular reliance on environmental P and Mg availability. While *Raphidonema* exists only at low abundance in snow^80^, *Microglena* is a dominant, blooming alga on snow^12^, and most likely requires such a strategy to successfully establish and maintain a bloom.

## Conclusion

Globally, our sc-ICP-ToF-MS data showed the diversity of elemental contents in single cells of cryosphere and mesophile algal species, providing a more in-depth evaluation of their ionomic profiles. Besides the mean elemental masses, this technique revealed the elemental mass distributions that, associated with the biovolume measurements, offered a better understanding of intra- and interspecies variations at a single-cell level. This level of cellular resolution opens novel perspectives on studies about algal metabolism and their interactions with their environment. Cellular nutrient status will not only be a by-product of nutrient availability, but also of external factors such as temperature, which will impact metabolic rates, liquid water availability, which will impact primary productivity (thus likely altering cellular Mg content), and light stress which will affect oxidative stress pathways (thus likely increasing the cellular requirements for trace metals such as Fe, Cu, and Mn). Investigating the impacts of light and temperature (including freezing) stress on terrestrial cryosphere algal ionomes is an important future research goal. Furthermore, little information has been reported about trace metal availability and use on glaciers and snow patches, highlighting a large knowledge gap for our understanding of cryosphere algae nutrient homeostasis and adaptation to extreme environments.

## Supplementary information

Transparent Peer Review file

Supplementary Information_Single-cell ionomes of terrestrial cryosphere algae

## Data Availability

The data that support the findings of this study are publicly available in GFZ Data Services with the following 10.5880/GFZ.YOGU.2026.001.
