# Supplementary material for: Single-cell ionomes of terrestrial cryosphere algae
Source: Commun Earth Environ. 2026 Jun 26;7(1):546. doi: 10.1038/s43247-026-03679-6 (PMC13309284; doi:10.1038/s43247-026-03679-6)
Supplement: Supplementary file 2 — Supplementary Information_Single-cell ionomes of terrestrial cryosphere algae [file 43247_2026_3679_MOESM2_ESM.pdf]

Supplementary Information for:

Single-cell ionomes of terrestrial cryosphere algae

Silvana R. Oliveira[1,2*&], Helen K. Feord[3*&], Cícero A. Lopes Júnior[4], Ravi S. Peters[3], Liane G. Benning[3,5] & Björn Meermann[1]

1. Federal Institute for Materials Research and Testing (BAM), Division 1.1 – Inorganic Trace Analysis (ITALab), 12489 Berlin, Germany

2. Department of Clinical Analysis, Toxicology and Food Sciences, School of Pharmaceutical Sciences of Ribeirão Preto, University of São Paulo (USP), 14040-903 Ribeirão Preto, Brazil

3. GFZ Helmholtz Center for Geosciences, 14473 Potsdam, Germany

4. Grupo de Estudos em Bioanalítica – GEBIO, Department of Chemistry, Federal University of Piauí, 64049-550 Teresina, Brazil

5. Department of Earth Sciences, Freie Universität Berlin, 12249 Berlin, Germany

*Corresponding authors: silvanaruella@usp.br and helen.feord@gfz.de

[&]These authors contributed equally

Supplementary Table 1. Media composition for the 4 media recipes used, analysed by ICP-MS, except for K which was analysed by ICP-OES

Element	Basal		3N BBM		TAP		TP	
	Mean (mg/L)	StDev	Mean (mg/L)	StDev	Mean (mg/L)	StDev	Mean (mg/L)	StDev
**Mg**	2.85	0.67	7.49	0.60	11.01	0.04	10.96	0.56
**P**	4.11	0.00	53.20	0.28	75.50	0.55	74.89	0.42
**Ca**	1.84	1.69	4.34	0.48	8.84	0.08	9.20	0.80
**Mn**	0.06	0.00	0.39	0.02	1.59	0.00	1.55	0.00
**Fe**	0.84	0.02	1.06	0.12	1.17	0.07	1.09	0.03
**Co**	Below LOQ*		0.10	0.00	0.45	0.00	0.44	0.00
**Cu**	Below LOQ*		0.40	0.01	0.49	0.03	0.52	0.03
**Mo**	Below LOQ*		0.37	0.01	0.62	0.00	0.60	0.00
**Zn**	Below LOQ*		2.09	0.06	6.02	0.06	5.86	0.06
**K**	67.39	1.36	82.45	1.37	115.11	1.26	115.33	0.94

*Limit of Quantification (LOQ) in μg L^{-1}: Mg 0.154; P 0.532; Ca 0.379; Mn 0.033; Fe 0.072; Co 0.026; Cu 0.059; Mo 0.088 and Zn 0.178;

Supplementary Table 2. Optimized operating conditions of the ICP-ToF-MS for single-cell analyses

ICP-ToF-MS parameters	Parameter Value
Plasma Power (W)	1550
Auxiliary gas flow (L min^{-1})	0.65
Cool gas flow (L min^{-1})	14
Nebulizer gas flow (L min^{-1})	0.29
Make-up gas flow (mL min^{-1})	650
CCT flow – H_2 (mL min^{-1})	3.00
Sampling depth (mm)	8.30
Sample flow rate (μL min^{-1})	10
Integration time (ms)	3
Measurement time (s)	300
Assessed isotopes	^{197}Au, ^{40}Ca, ^{63}Cu, ^{56}Fe, ^{24}Mg, ^{24}Mn, ^{31}P, ^{64}Zn

Supplementary Table 3. LOD and LOQ (fg cell^{-1}) for the 7 analyzed elements using sc-ICP-ToF-MS.

Element	LOD (fg cell^{-1})*	LOQ (fg cell^{-1})**
Mg	0.20	0.68
P	2.60	8.68
Ca	0.17	0.57
Cu	0.04	0.14
Fe	0.35	1.16
Mn	0.06	0.21
Zn	0.09	0.33

*Limits of Detection (in fg cell^{-1}) were calculated using the equation proposed for analog to digital conversion signals by Gundlach−Graham et al. 2018 and the intensities were converted into the mass of elements by transformed slope (in counts/g) of the calibration curve.
**Limits of Quantification (in fg cell^{-1}) were calculated as 3.3*LOD.

Supplementary Table 4. Phosphorous contents (fg cell^{-1}) for biological triplicate analyses of single cells of the three cryosphere chlorophyte snow algae and the two mesophile chlorophyte algae. Culture medium used for each algal species is given in brackets.

Sample	Biological replicate	P Concentration (fg cell^{-1})				
		Minimum	Maximum	Mean	Median	25 - 75 percentile
***Microglena sp.* (3N-BBM)**	1	159.28	15950.74	2159.50	1755.45	1124.79 - 2723.89
	1	168.09	15173.28	2209.21	1814.51	1136.20 – 2788.32
	2	165.21	7277.91	863.63	556.66	367.08 – 1006.22
	2	179.64	8947.74	770.40	517.98	342.70 – 928.85
	3	166.62	21862.63	1061.58	667.98	363.88 – 1259.86
	3	152.56	15458.61	1128.81	646.14	366.94 – 1128.81
***Raphidonema sempervirens* (3N-BBM)**	1	146.64	5786.33	664.49	532.62	344.57 – 827.96
	1	150.77	3943.78	688.91	559.97	355.46 – 864.57
	1	149.08	4192.82	684.24	544.86	342.58 – 864.13
	2	92.18	7976.28	1204.73	1033.59	677.77 – 1554.67
	2	106.19	4883.83	1243.05	1063.18	704.66 – 1586.01
	2	116.94	7158.99	1252.23	1047.59	648.53 – 1624.96
	3	169.38	6051.14	697.85	573.41	378.69 – 866.25
	3	169.04	3782.43	683.06	567.54	373.40 – 887.78
	3	172.31	4540.24	689.25	564.99	381.85 – 869.05
***Deuterostichococcus sp.* (3N-BBM)**	1	87.82	3537.88	435.13	345.66	221.85 – 548.93
	1	88.79	2715.08	407.39	326.85	213.16 – 510.60
	1	90.28	2748.74	410.85	328.76	211.39 – 513.22
	2	90.14	2653.74	464.37	388.52	257.18 - 595.85
	2	87.37	2549.40	445.82	381.06	245.41 – 574.86
	2	89.25	4047.75	457.67	382.00	253.41 – 584.70
	3	89.81	2987.45	374.61	303.01	199.67 – 468.80
	3	89.41	4623.28	359.70	291.52	193.30 – 448.97
	3	87.45	2817.16	365.54	296.49	195.96 – 458.64
***Chlamydomonas reinhardti* (TP)**	1	82.37	19448.08	2428.72	1342.30	407.16 – 3757.37
	1	84.15	16453.27	2070.32	917.71	380.62 – 3017.58
	1	88.59	17667.64	2930.81	2043.62	503.08 – 4516.60
	2	104.80	23639.08	3475.61	2423.95	813.12 – 5178.18
	2	110.77	28426.90	2752.34	1470.33	475.19 – 3763.63
	2	108.58	23422.90	3640.38	2395.74	781.28 – 5365.07
	3	106.25	17746.32	2846.38	2264.04	1203.07 – 3870.90
	3	111.77	20838.98	2506.66	1857.69	769.43 – 3506.01
	3	110.82	13664.89	2371.36	1753.28	685.67 – 3368.91
***Chlamydomonas reinhardti* (TAP)**	1	108.00	9252.65	1360.55	1166.82	760.41 – 1743.94
	1	111.30	11233.33	1420.60	1196.10	771.08 – 1830.77
	1	103.72	10730.68	1338.27	1137.56	750.84 – 1694.17
	2	106.45	9188.68	1414.51	1196.64	698.59 – 1878.45
	2	106.78	7606.61	1347.55	1111.09	683.13 – 1815.35
	2	108.23	6673.45	1302.31	1082.26	647.23 – 1718.50
	3	104.52	10617.56	1377.63	1115.08	705.16 – 1736.98
	3	102.95	8899.31	1161.53	944.82	459.49 – 1545.11
	3	101.64	7943.23	1213.39	1008.37	511.17 – 1603.04
***Acutodesmus obliquus* (Basal)**	1	83.31	2444.14	476.73	393.92	265.98 – 607.99
	1	84.68	2801.56	522.50	419.07	272.52 – 655.17
	1	80.66	3003.13	520.29	426.75	288.77 – 653.30
	2	86.96	2719.36	407.93	336.27	209.61 – 517.77
	2	88.03	3865.70	421.18	337.77	220.65 – 531.38
	2	84.15	6164.97	418.35	341.10	216.14 – 522.23
	3	88.53	2617.13	479.72	399.77	260.60 – 616.25
	3	90.21	3866.29	492.56	400.71	262.28 – 615.83
	3	87.80	2998.11	463.42	382.83	256.56 – 589.24

Supplementary Table 5. Magnesium contents (fg cell^{-1}) for biological triplicate analyses of single cells of the three cryosphere chlorophyte snow algae and the two mesophile chlorophyte algae. Culture medium used for each algal species is given in brackets.

Sample	Biological replicate	Mg Concentration (fg cell^{-1})				
		Minimum	Maximum	Mean	Median	25 - 75 percentile
***Microglena sp.* (3N-BBM)**	1	17.71	1909.06	356.88	302.18	212.20 – 448.79
	1	17.57	1657.44	371.19	319.63	224.35 – 478.28
	2	19.51	640.23	112.68	83.69	46.87 – 140.98
	2	14.60	1184.34	103.41	75.53	44.44 – 128.99
	3	20.15	2167.27	165.67	116.44	65.52 – 197.56
	3	13.92	1493.00	169.02	111.71	60.90 – 220.27
***Raphidonema sempervirens* (3N-BBM)**	1	10.06	355.75	54.96	45.68	28.46 – 72.22
	1	9.99	262.91	54.30	44.10	28.14 – 70.66
	1	9.63	229.60	53.26	43.70	27.50 – 68.95
	2	5.63	339.53	94.29	84.33	54.05 – 127.54
	2	5.13	416.53	94.76	87.20	55.30 – 125.89
	2	5.46	545.56	94.82	85.07	51.09 – 125.10
	3	11.71	452.56	58.06	52.47	35.08 – 74.09
	3	11.94	364.62	58.66	52.95	35.61 – 74.04
	3	11.63	281.61	57.53	51.77	34.51 – 73.97
***Deuterostichococcus sp.* (3N-BBM)**	1	8.54	310.26	48.40	40.72	28.81 – 59.74
	1	8.40	288.09	46.68	40.07	28.57 – 57.39
	1	7.79	274.32	47.56	40.75	28.78 – 57.84
	2	8.27	212.80	47.79	42.21	31.00 – 58.81
	2	7.60	223.49	48.76	43.49	32.38 – 59.19
	2	8.17	232.63	49.21	43.73	32.40 – 59.85
	3	8.94	230.35	48.12	41.87	29.99 – 58.92
	3	8.76	246.20	48.50	42.41	30.62 – 59.20
	3	8.83	247.91	49.62	43.41	31.02 – 61.15
***Chlamydomonas reinhardti* (TP)**	1	5.56	1401.67	235.79	145.40	36.92 – 394.18
	1	6.90	1221.67	188.77	84.35	33.65 – 302.57
	1	6.46	1155.53	271.00	218.73	46.29 – 454.46
	2	7.04	1645.86	327.12	255.22	82.09 – 508.56
	2	6.89	1609.32	254.20	156.24	43.42 – 367.84
	2	6.85	1575.26	322.31	240.87	68.56 – 498.71
	3	6.17	1496.91	309.03	276.59	150.73 – 429.67
	3	7.29	1841.65	253.70	203.96	83.62 – 375.61
	3	6.34	1107.81	243.16	195.24	75.75 – 363.59
***Chlamydomonas reinhardti* (TAP)**	1	7.40	970.77	219.81	204.12	141.38 – 279.95
	1	6.77	1249.36	217.15	195.57	132.64 – 268.26
	1	6.13	1179.08	203.74	180.25	125.81 – 256.75
	2	6.17	874.22	193.09	184.87	81.73 – 270.46
	2	6.40	883.51	186.07	181.24	69.68 – 262.68
	2	6.91	871.16	175.33	167.11	65.95 – 254.47
	3	6.02	794.59	130.18	119.64	76.54 – 161.51
	3	6.62	800.68	108.07	103.73	39.71 – 149.23
	3	6.31	633.49	107.25	100.79	40.22 – 147.50
***Acutodesmus obliquus* (Basal)**	1	6.26	463.32	118.24	107.22	79.27 – 147.59
	1	19.08	546.68	129.68	116.10	84.91 – 161.09
	1	7.34	676.52	127.50	115.52	85.21 – 157.07
	2	5.24	686.94	116.74	107.31	75.19 – 144.95
	2	8.52	856.66	118.96	109.13	75.58 – 149.43
	2	6.51	549.37	119.70	110.64	76.37 – 150.02
	3	5.42	667.19	117.49	106.81	77.84 – 146.01
	3	7.69	423.84	118.10	108.13	79.50 – 145.71
	3	7.79	600.93	114.45	104.50	76.61 – 141.14

Supplementary Table 6. Calcium contents (fg cell^{-1}) for biological triplicate analyses of single cells of the three cryosphere chlorophyte snow algae and the two mesophile chlorophyte algae. Culture medium used for each algal species is given in brackets.

Sample	Biological replicate	Ca Concentration (fg cell^{-1})				
		Minimum	Maximum	Mean	Median	25 - 75 percentile
***Microglena sp.* (3N-BBM)**	1	1.73	177.20	31.69	29.13	11.88 – 44.02
	1	1.73	180.77	31.55	28.34	11.10 – 44.40
	2	4.48	428.54	61.33	44.05	19.90 – 85.75
	2	3.87	383.39	57.69	43.22	18.98 – 77.84
	3	3.04	533.94	48.27	29.86	10.74 – 63.31
	3	3.50	398.16	48.33	33.67	13.74 – 61.34
***Raphidonema sempervirens* (3N-BBM)**	1	2.28	94.92	15.94	13.61	6.16 – 21.53
	1	2.24	74.54	14.62	12.33	4.91 – 20.17
	1	2.26	118.48	15.62	13.71	5.24 – 21.29
	2	1.25	484.87	17.09	6.37	4.09 – 10.53
	2	1.31	416.41	18.40	6.67	4.34 – 10.71
	2	1.20	338.57	17.52	6.02	3.57 – 9.62
	3	3.05	142.68	18.26	14.19	4.20 – 27.51
	3	3.02	134.53	18.89	11.70	4.09 – 28.75
	3	2.91	100.92	17.70	10.60	4.02 – 27.63
***Deuterostichococcus sp.* (3N-BBM)**	1	2.07	127.00	16.29	14.30	10.40 – 20.0
	1	2.74	575.05	19.18	16.88	12.49 – 23.26
	1	2.61	177.38	20.66	18.44	13.32 – 25.53
	2	2.26	186.96	22.72	19.82	14.21 – 28.05
	2	1.99	357.72	25.13	22.40	15.94 – 30.99
	2	1.93	150.33	24.38	21.89	15.66 – 30.32
	3	2.39	69.04	13.71	12.20	8.68 – 16.96
	3	2.24	76.75	15.66	14.09	10.18 – 19.30
	3	2.25	121.79	16.39	14.80	10.55 – 20.16
***Chlamydomonas reinhardti* (TP)**	1	1.63	1792.50	317.72	206.65	83.91 – 495.78
	1	2.25	1530.80	253.48	155.43	76.08 – 363.13
	1	1.63	1321.69	318.67	226.07	84.59 – 507.13
	2	2.01	1529.33	315.12	237.84	100.36 – 463.89
	2	3.30	1864.42	284.84	195.32	79.62 – 402.12
	2	7.38	1616.15	329.47	257.20	100.28 – 484.61
	3	2.07	1804.58	298.60	263.26	125.79 – 429.63
	3	3.44	1753.05	294.18	226.97	101.60 – 431.69
	3	2.31	1255.17	246.78	191.63	82.81 – 367.54
***Chlamydomonas reinhardti* (TAP)**	1	1.20	4103.14	84.51	80.03	47.98 – 114.93
	1	3.13	379.71	87.76	82.96	49.57 – 116.62
	1	1.25	407.90	85.27	81.54	49.13 – 113.13
	2	1.16	356.93	67.44	53.87	31.89 – 88.72
	2	1.04	422.73	66.72	53.83	31.82 – 87.19
	2	1.18	355.38	64.21	52.58	29.70 – 86.93
	3	0.94	669.53	134.27	123.68	81.93 – 167.51
	3	1.37	638.78	118.89	110.23	59.08 – 160.24
	3	1.42	564.51	121.41	115.38	64.10 – 157.74
***Acutodesmus obliquus* (Basal)**	1	0.68	83.16	12.14	10.49	5.75 – 16.30
	1	0.77	72.59	13.88	11.78	6.54 – 18.75
	1	0.75	98.53	13.80	11.90	6.68 – 18.64
	2	0.75	132.60	9.22	7.33	3.94 – 12.12
	2	0.70	119.72	9.46	7.54	3.97 – 12.09
	2	0.72	114.90	9.24	7.47	4.16 – 11.77
	3	0.80	391.72	16.25	14.73	9.15 – 21.42
	3	0.71	85.94	15.87	14.11	9.21 – 21.23
	3	0.79	118.14	15.48	13.99	8.97 – 20.22

Supplementary Table 7. Copper contents (fg cell^{-1}) for biological triplicate analyses of single cells of the three cryosphere chlorophyte snow algae and the two mesophile chlorophyte algae. Culture medium used for each algal species is given in brackets.

Sample	Biological replicate	Cu Concentration (fg cell^{-1})				
		Minimum	Maximum	Mean	Median	25 - 75 percentile
***Microglena sp.* (3N-BBM)**	1	0.61	41.32	3.88	2.54	1.26 – 5.50
	1	0.63	32.24	3.81	2.52	1.24 – 5.16
	2	0.49	102.86	10.11	7.72	4.41 – 13.22
	2	0.84	66.23	11.31	8.10	3.83 – 14.94
	3	0.88	103.40	9.65	7.63	4.01 – 12.83
	3	0.77	81.48	11.06	8.96	4.51 – 14.49
***Raphidonema sempervirens* (3N-BBM)**	1	0.66	6.74	1.03	0.87	0.77 – 1.05
	1	0.69	62.57	1.50	0.92	0.80 – 1.16
	1	0.67	2.92	1.01	0.88	0.79 – 1.11
	2	0.51	4.80	1.15	0.95	0.70 – 1.41
	2	0.52	4.10	1.14	0.94	0.71 – 1.39
	2	0.52	5.16	1.12	0.90	0.69 – 1.30
	3	0.75	3.11	1.06	0.95	0.83 – 1.16
	3	0.74	3.73	1.03	0.92	0.83 – 1.07
	3	0.73	3.90	1.10	0.94	0.82 – 1.16
***Deuterostichococcus sp.* (3N-BBM)**	1	0.59	1.32	0.78	0.74	0.68 – 0.83
	1	0.60	1.37	0.79	0.75	0.70 – 0.85
	1	0.61	7.13	0.83	0.74	0.69 – 0.86
	2	0.56	0.97	0.71	0.69	0.63 – 0.77
	2	0.56	1.19	0.71	0.67	0.62- 0.78
	2	0.57	1.22	0.71	0.67	0.61 – 0.75
	3	0.60	15.44	1.13	0.79	0.71 – 0.97
	3	0.60	72.61	1.36	0.80	0.71 – 0.96
	3	0.59	12.28	0.93	0.78	0.71 – 0.89
***Chlamydomonas reinhardti* (TP)**	1	0.59	11.15	1.55	1.34	0.99 – 1.87
	1	0.53	5.07	1.44	1.26	0.93 – 1.78
	1	0.56	5.42	1.41	1.22	0.87 – 1.76
	2	0.56	4.42	1.20	1.03	0.79 – 1.46
	2	0.56	10.45	1.37	1.17	0.84 – 1.63
	2	0.56	5.18	1.42	1.22	0.89 – 1.73
	3	0.54	6.27	1.05	0.95	0.78 – 1.20
	3	0.58	4.93	1.26	1.10	0.85 – 1.49
	3	0.55	3.01	1.00	0.88	0.75 – 1.15
***Chlamydomonas reinhardti* (TAP)**	1	0.52	2.81	0.92	0.83	0.72 – 1.03
	1	0.56	5.67	0.97	0.85	0.73 – 1.05
	1	0.56	3.21	0.95	0.84	0.72 – 1.06
	2	0.56	20.92	2.49	1.51	0.98 – 2.89
	2	0.57	28.41	2.57	1.53	0.98 – 3.16
	2	0.54	34.41	2.47	1.47	0.97 – 2.84
	3	0.57	6.06	1.30	1.11	0.85 – 1.52
	3	0.55	6.47	1.26	1.13	0.83 – 1.52
	3	0.55	9.73	1.23	1.08	0.83 – 1.46
***Acutodesmus obliquus* (Basal)**	1	0.55	0.97	0.72	0.70	0.66 – 0.79
	1	0.53	1.15	0.67	0.63	0.58 – 0.72
	1	0.50	1.39	0.68	0.63	0.58 – 0.71
	2	0.58	1.28	0.70	0.66	0.63 – 0.72
	2	0.57	0.88	0.68	0.67	0.61 – 0.73
	2	0.59	0.84	0.66	0.65	0.62 – 0.69
	3	0.55	1.41	0.72	0.68	0.60 – 0.75
	3	0.57	1.22	0.71	0.66	0.61 – 0.76
	3	0.56	1.07	0.71	0.68	0.63 – 0.77

Supplementary Table 8. Iron contents (fg cell^{-1}) for biological triplicate analyses of single cells of the three cryosphere chlorophyte snow algae and the two mesophile chlorophyte algae. Culture medium used for each algal species is given in brackets.

Sample	Biological replicate	Fe Concentration (fg cell^{-1})				
		Minimum	Maximum	Mean	Median	25 - 75 percentile
	1	6.66	827.33	111.90	76.68	49.54 – 154.58
	1	6.11	782.47	113.08	77.66	49.77 – 156.46
Microglena sp.	2	9.17	4729.46	165.72	63.57	20.00 – 105.92
**(3N-BBM)**	2	9.14	3805.79	181.26	49.38	17.70 – 151.55
	3	6.97	1198.10	82.18	60.51	26.69 – 88.71
	3	7.56	1206.33	91.82	66.65	45.55 – 94.92
	1	5.47	95.48	15.08	12.84	9.48 – 18.13
	1	5.36	124.80	15.08	12.88	9.32 – 18.36
	1	5.33	89.09	14.87	12.96	9.46 – 18.17
Raphidonema	2	4.24	104.03	12.32	10.91	7.78 – 15.16
sempervirens	2	4.05	50.67	12.19	11.21	7.75 – 15.27
**(3N-BBM)**	2	3.98	94.84	12.64	11.11	7.80 – 15.69
	3	14.02	2738.99	54.27	35.04	24.90 – 54.98
	3	14.25	1264.66	49.05	35.18	24.57 – 54.61
	3	14.85	2501.04	50.36	34.64	24.37 – 54.32
	1	3.82	456.37	17.24	14.76	10.32 – 21.19
	1	4.46	2002.08	17.46	14.80	10.35 – 21.40
	1	4.43	3450.67	17.29	14.57	10.12 – 20.72
Deuterostichococcus	2	4.79	136.85	32.53	28.86	20.13 – 40.98
***sp.* (3N-BBM)**	2	4.53	202.43	31.89	28.60	20.06 – 40.07
	2	4.15	142.75	32.02	28.76	19.72 – 40.27
	3	4.57	102.71	20.03	17.64	12.42 – 24.66
	3	4.31	85.40	19.49	17.27	12.17 – 24.14
	3	4.28	164.41	20.19	17.83	12.50 – 24.88
	1	4.35	861.44	89.50	79.96	24.60 – 134.30
	1	3.94	704.72	76.15	60.68	15.80 – 120.12
	1	4.42	738.25	91.45	83.61	35.53 – 134.18
Chlamydomonas	2	4.40	445.64	107.27	91.32	46.86 – 162.97
***reinhardti* (TP)**	2	4.23	6428.79	104.72	70.36	22.23 – 148.53
	2	4.38	739.07	127.26	101.07	45.43 – 186.43
	3	4.73	434.13	104.15	90.99	59.78 – 138.93
	3	4.40	1035.25	123.99	104.08	52.98 – 172.63
	3	4.37	441.22	91.18	78.12	43.65 – 127.18
	1	4.66	726.91	86.69	77.70	58.96 – 100.89
	1	4.77	634.08	91.37	82.14	61.33 – 108.87
	1	4.91	575.75	85.38	76.49	57.12 – 100.37
Chlamydomonas	2	4.53	1050.31	118.37	114.99	58.59 – 157.29
***reinhardti* (TAP)**	2	4.98	1784.78	118.82	114.06	58.12 – 158.19
	2	4.98	627.55	112.03	110.56	55.29 – 153.61
	3	4.66	381.69	83.76	76.47	52.36 – 103.79
	3	4.98	400.20	71.21	67.45	30.43 – 95.61
	3	5.14	405.39	76.37	71.48	36.56 – 101.34
	1	5.42	1523.02	46.10	37.44	26.25 – 52.18
	1	6.59	1095.01	48.15	41.39	29.44 – 58.61
	1	4.96	1221.22	47.97	40.95	29.25 – 58.42
Acutodesmus	2	5.28	359.28	49.77	45.13	32.00 – 62.45
***obliquus* (Basal)**	2	4.53	98.38	51.41	47.08	32.72 – 64.79
	2	5.01	450.37	50.33	46.48	32.58 – 62.50
	3	5.52	611.31	48.52	43.21	32.56 – 58.10
	3	5.51	422.05	47.45	42.83	31.52 – 57.69
	3	7.23	257.03	45.13	41.05	30.17 – 55.22

Supplementary Table 9. Manganese contents (fg $cell^{-1}$) for biological triplicate analyses of single cells of the cryosphere chlorophyte snow algae and the two mesophile chlorophyte algae. Culture medium used for each algal species is given in brackets.

Sample	Biological replicate	Mn Concentration (fg $cell^{-1}$)				
		Minimum	Maximum	Mean	Median	25 - 75 percentile
***Microglena sp.* (3N-BBM)**	1	0.50	96.13	6.85	5.64	3.82 – 8.57
	1	0.52	42.20	7.30	6.23	4.20 – 9.32
	2	0.48	35.81	5.64	3.92	2.19 – 7.28
	2	0.57	51.15	5.78	4.10	2.53 – 7.18
	3	0.83	84.64	6.82	4.28	2.44 – 8.29
	3	0.54	75.78	8.85	5.40	3.04 – 10.97
***Raphidonema sempervirens* (3N-BBM)**	1	0.48	19.49	2.16	1.81	1.17 – 2.71
	1	0.49	33.50	2.25	1.84	1.20 – 2.78
	1	0.50	15.91	2.23	1.87	1.21 – 2.80
	2	0.26	29.71	2.68	2.16	1.38 – 3.34
	2	0.27	17.73	2.66	2.17	1.37 – 3.18
	2	0.26	20.10	2.40	1.88	1.20 – 3.85
	3	0.65	22.30	3.37	2.80	1.90 – 4.09
	3	0.68	23.83	3.24	2.74	1.87 – 4.00
	3	0.65	17.63	3.21	2.72	1.89 – 3.99
***Deuterostichococcus sp.* (3N-BBM)**	1	0.29	5.04	1.09	0.94	0.67 – 1.34
	1	0.32	5.28	1.14	1.00	0.71 – 1.40
	1	0.31	15.64	1.13	0.99	0.69 – 1.40
	2	0.31	17.92	0.99	0.85	0.62 – 1.19
	2	0.29	14.36	0.96	0.84	0.60 – 1.17
	2	0.31	6.48	0.91	0.79	0.58 – 1.10
	3	0.30	5.82	1.27	1.13	0.79 – 1.58
	3	0.31	6.40	1.24	1.10	0.78 – 1.55
	3	0.30	8.01	1.28	1.13	0.80 – 1.60
***Chlamydomonas reinhardti* (TP)**	1	0.45	257.87	38.44	25.25	6.22 – 63.84
	1	0.39	204.71	29.54	12.44	5.09 – 49.98
	1	0.39	189.68	39.16	33.70	6.41 – 63.82
	2	0.44	169.72	45.15	38.27	10.67 – 76.02
	2	0.58	271.21	39.78	25.36	6.83 – 60.07
	2	0.53	204.70	47.79	39.68	10.01 – 76.88
	3	0.40	147.42	27.88	25.53	15.39 – 38.26
	3	0.38	203.84	29.51	26.02	8.92 – 42.16
	3	0.39	124.03	22.45	20.65	6.32 – 32.60
***Chlamydomonas reinhardti* (TAP)**	1	0.32	26.86	4.57	4.28	3.33 – 5.46
	1	0.29	25.56	4.71	4.39	3.38 – 5.67
	1	0.29	22.28	4.45	4.12	3.13 – 5.32
	2	0.27	24.83	5.55	5.48	2.38 – 7.89
	2	0.27	23.45	5.35	5.36	2.23 – 7.67
	2	0.27	18.93	5.15	5.10	2.10 – 7.54
	3	0.32	26.99	4.66	4.30	2.95 – 5.55
	3	0.29	22.11	3.98	3.82	1.81 – 5.23
	3	0.28	23.59	3.97	3.74	2.00 – 5.15
***Acutodesmus obliquus* (Basal)**	1	0.26	25.89	1.85	1.62	1.11 – 2.28
	1	0.27	19.77	2.09	1.81	1.26 – 2.62
	1	0.26	19.81	2.07	1.82	1.24 – 2.63
	2	0.26	14.83	1.91	1.69	1.09 – 2.41
	2	0.24	22.69	1.96	1.71	1.12 – 2.43
	2	0.24	27.60	1.95	1.68	1.09 – 2.45
	3	0.25	14.17	2.19	1.99	1.41 – 2.73
	3	0.26	32.28	2.18	1.96	1.36 – 2.73
	3	0.26	10.38	2.08	1.89	1.32 – 2.61

Supplementary Table 10. Zinc contents (fg $cell^{-1}$) for biological triplicate analyses of single cells of the three cryosphere chlorophyte snow algae and the two mesophile chlorophyte algae. Culture medium used for each algal species is given in brackets.

Sample	Biological replicate	Zn Concentration (fg $cell^{-1}$)				
		Minimum	Maximum	Mean	Median	25 - 75 percentile
***Microglena sp.* (3N-BBM)**	1	0.99	38.82	5.89	4.53	2.83 – 7.28
	1	1.06	40.36	6.36	4.92	3.13 – 7.97
	2	1.35	131.16	4.63	3.11	2.30 – 5.06
	2	1.51	44.63	4.74	3.53	2.38 – 5.46
	3	1.17	159.24	3.65	2.30	1.85 – 3.24
	3	1.33	39.71	4.16	2.95	1.97 – 4.71
***Raphidonema sempervirens* (3N-BBM)**	1	0.94	7.55	1.55	1.41	1.18 – 1.73
	1	0.96	9.02	1.65	1.44	1.21 – 1.93
	1	1.01	8.81	1.67	1.51	1.25 – 1.85
	2	0.70	13.94	2.06	1.66	1.21 – 2.37
	2	0.76	24.94	2.52	1.74	1.22 – 2.47
	2	0.72	28.43	2.53	1.66	1.18 – 2.43
	3	1.26	8.20	2.26	1.95	1.64 – 2.56
	3	1.25	8.41	2.24	2.03	1.69 – 2.56
	3	1.23	74.39	2.32	1.97	1.67 – 2.50
***Deuterostichococcus sp.* (3N-BBM)**	1	0.87	111.17	2.31	2.01	1.74 – 2.47
	1	1.21	12.20	2.16	1.94	1.67 - 2.40
	1	1.19	13.33	2.19	1.95	1.64 – 2.43
	2	1.07	13.18	2.77	2.40	1.83 – 3.34
	2	0.96	20.37	2.72	2.38	1.72 – 3.32
	2	0.97	17.19	2.69	2.31	1.72 – 3.21
	3	1.09	6.65	1.88	1.70	1.47 – 2.07
	3	1.00	21.83	1.83	1.65	1.41 – 2.03
	3	1.07	17.16	1.89	1.68	1.45 – 2.08
***Chlamydomonas reinhardti* (TP)**	1	1.12	79.00	15.41	15.06	4.33 – 23.50
	1	0.91	65.37	12.99	8.75	3.33 – 21.33
	1	0.88	70.79	15.70	15.39	4.79 – 23.50
	2	1.03	62.82	6.97	6.41	3.70 – 9.79
	2	1.15	257.50	8.19	6.25	3.34 – 11.02
	2	1.23	31.76	8.74	7.30	4.17 – 12.12
	3	1.06	176.90	4.18	3.32	2.24 – 4.84
	3	1.08	816.99	5.34	3.90	2.55 – 5.96
	3	1.00	257.94	3.48	2.78	1.90 – 4.08
***Chlamydomonas reinhardti* (TAP)**	1	0.81	9.01	2.37	2.00	1.39 – 2.90
	1	0.92	15.53	3.32	2.62	1.72 – 4.30
	1	0.90	25.78	3.51	2.87	1.93 – 4.57
	2	0.71	7.82	1.81	1.60	1.16 – 2.22
	2	0.75	6.23	1.89	1.66	1.20 – 2.34
	2	0.81	7.32	1.88	1.69	1.24 – 2.26
	3	0.70	19.42	1.69	1.40	1.08 – 1.95
	3	0.71	6.56	1.65	1.45	1.11 – 1.91
	3	0.74	11.84	1.81	1.51	1.16 – 2.06
***Acutodesmus obliquus* (Basal)**	1	0.71	3.09	1.18	1.09	0.89 – 1.35
	1	0.84	4.08	1.34	1.21	1.06 – 1.48
	1	0.82	4.30	1.33	1.20	1.02 – 1.52
	2	0.68	5.09	1.27	1.14	0.94 – 1.44
	2	0.69	3.51	1.29	1.16	0.95 – 1.48
	2	0.70	5.09	1.28	1.15	0.94 – 1.49
	3	0.66	6.20	1.24	1.10	0.89 – 1.43
	3	0.65	8.21	1.24	1.10	0.90 – 1.44
	3	0.68	9.63	1.21	1.09	0.89 – 1.38

Supplementary Table 11. Statistics (PERMANOVA + t-test) of the effect of P-starvation on *Microglena sp*. cellular elemental concentrations.

Replicate	group	element	Mean (g/cell)	Percentage change of means(%)	PERMANOVA p value	Unpaired t-test p value
1	P_replete	Ca	4.22E-14	-48.83	0.001	0.012
1	P_starved		2.16E-14			
1	P_replete	Cu	1.66E-15	84.08	0.001	0.011
1	P_starved		3.06E-15			
1	P_replete	Fe	5.24E-14	11.84	0.01	>0.5
1	P_starved		5.86E-14			
1	P_replete	Mg	1.66E-13	-73.48	0.001	0.012
1	P_starved		4.40E-14			
1	P_replete	Mn	3.67E-15	3.04	>0.5	>0.5
1	P_starved		3.78E-15			
1	P_replete	P	7.82E-13	-53.19	0.001	0.000626
1	P_starved		3.66E-13			
1	P_replete	Zn	2.70E-15	-0.30	>0.5	>0.5
1	P_starved		2.70E-15			
2	P_replete	Ca	9.54E-14	-11.37	0.001	>0.5
2	P_starved		8.46E-14			
2	P_replete	Cu	1.59E-15	79.28	0.001	0.025
2	P_starved		2.85E-15			
2	P_replete	Fe	1.17E-13	10.08	0.001	>0.5
2	P_starved		1.28E-13			
2	P_replete	Mg	5.11E-13	-80.39	0.001	0.003
2	P_starved		1.00E-13			
2	P_replete	Mn	8.01E-15	7.12	0.02	>0.5
2	P_starved		8.58E-15			
2	P_replete	P	2.17E-12	-59.46	0.001	0.000814
2	P_starved		8.79E-13			
2	P_replete	Zn	1.12E-14	-22.26	0.001	>0.5
2	P_starved		8.74E-15			

Supplementary Table 12. Statistics (PERMANOVA + t-test) of the effect of P-starvation on *Raphidonema sempervirens* cellular elemental concentrations.

Replicate	group	element	mean	Percentage change (%)	PERMANOVA p value	unpaired t-test p value
1	P_replete	Ca	1.74E-14	7.36	0.027	>0.5
1	P_starved		1.87E-14			
1	P_replete	Cu	1.73E-15	-34.09	0.001	>0.5
1	P_starved		1.14E-15			
1	P_replete	Fe	4.39E-13	-42.22	0.001	>0.5
1	P_starved		2.54E-13			
1	P_replete	Mg	5.05E-14	5.25	0.001	>0.5
1	P_starved		5.32E-14			
1	P_replete	Mn	2.85E-15	27.53	0.001	>0.5
1	P_starved		3.64E-15			
1	P_replete	P	4.64E-13	-14.61	0.001	>0.5
1	P_starved		3.96E-13			
1	P_replete	Zn	1.99E-15	-8.96	0.001	>0.5
1	P_starved		1.81E-15			
2	P_replete	Ca	2.60E-14	-2.26	>0.5	>0.5
2	P_starved		2.54E-14			
2	P_replete	Cu	9.48E-16	-7.79	0.002	>0.5
2	P_starved		8.74E-16			
2	P_replete	Fe	3.57E-14	-6.52	0.003	0.007
2	P_starved		3.33E-14			
2	P_replete	Mg	8.69E-14	-21.90	0.001	0.049
2	P_starved		6.79E-14			
2	P_replete	Mn	3.03E-15	-28.17	0.001	0.018
2	P_starved		2.18E-15			
2	P_replete	P	7.28E-13	-22.97	0.001	0.002
2	P_starved		5.61E-13			
2	P_replete	Zn	5.26E-15	48.33	0.001	>0.5
2	P_starved		7.80E-15			

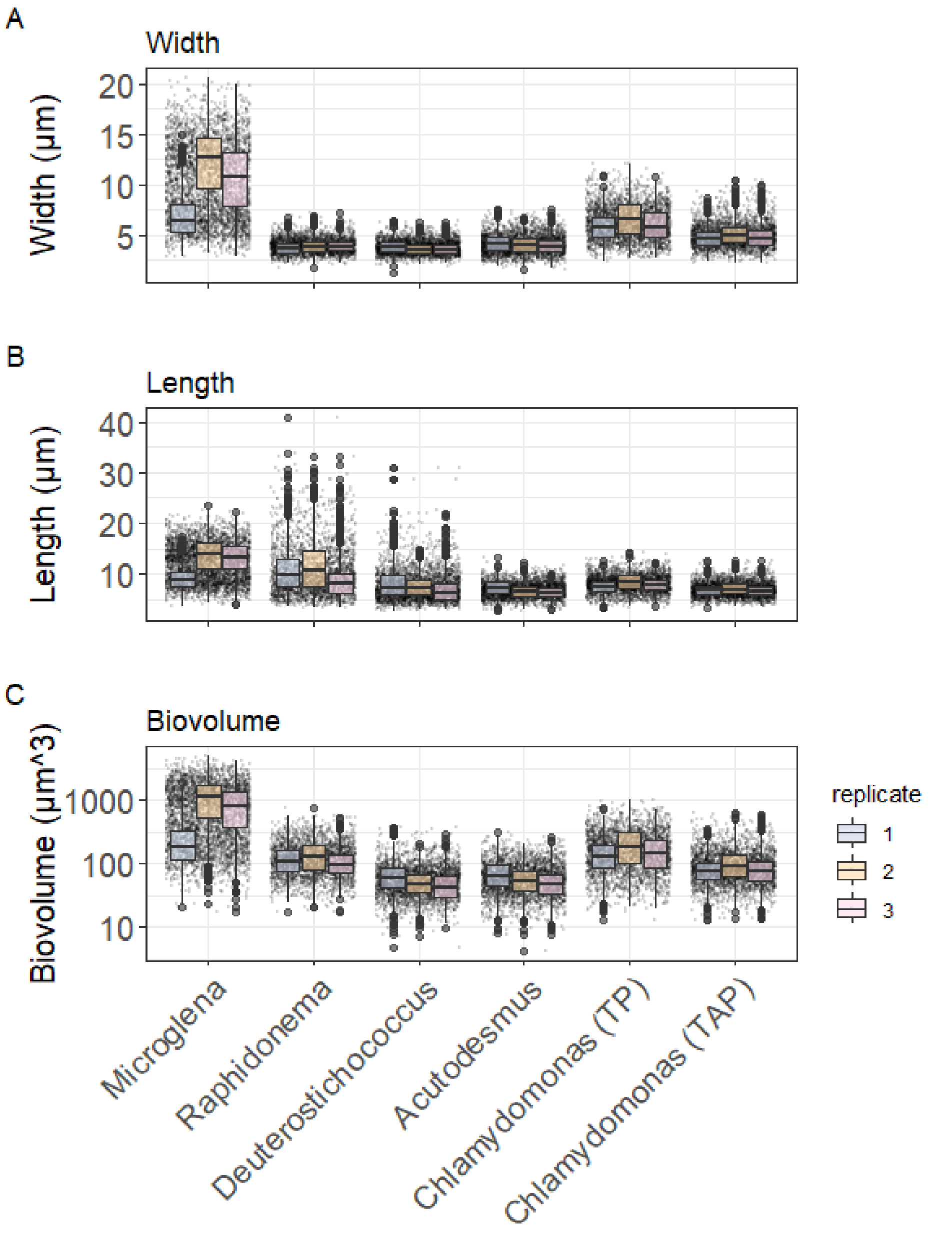

Supplementary Figure 1. Calculated cellular width, length, and biovolume for each culture. Box plots for **(A)** width, **(B)** length, and **(C)** biovolume of the cells from all algae species analysed (biological replicates combined) – cryosphere (*Microglena sp.*, *Raphidonema sempervirens, Deuterostichococcus sp.*) and mesophile chlorophyte species (*Chlamydomonas reinhardtii* - grown in TAP and TP media and *Acutodesmus obliquus).* The boxes begin with the first quartile and ends with the third quartile, with the median indicated as a line that divides the box.

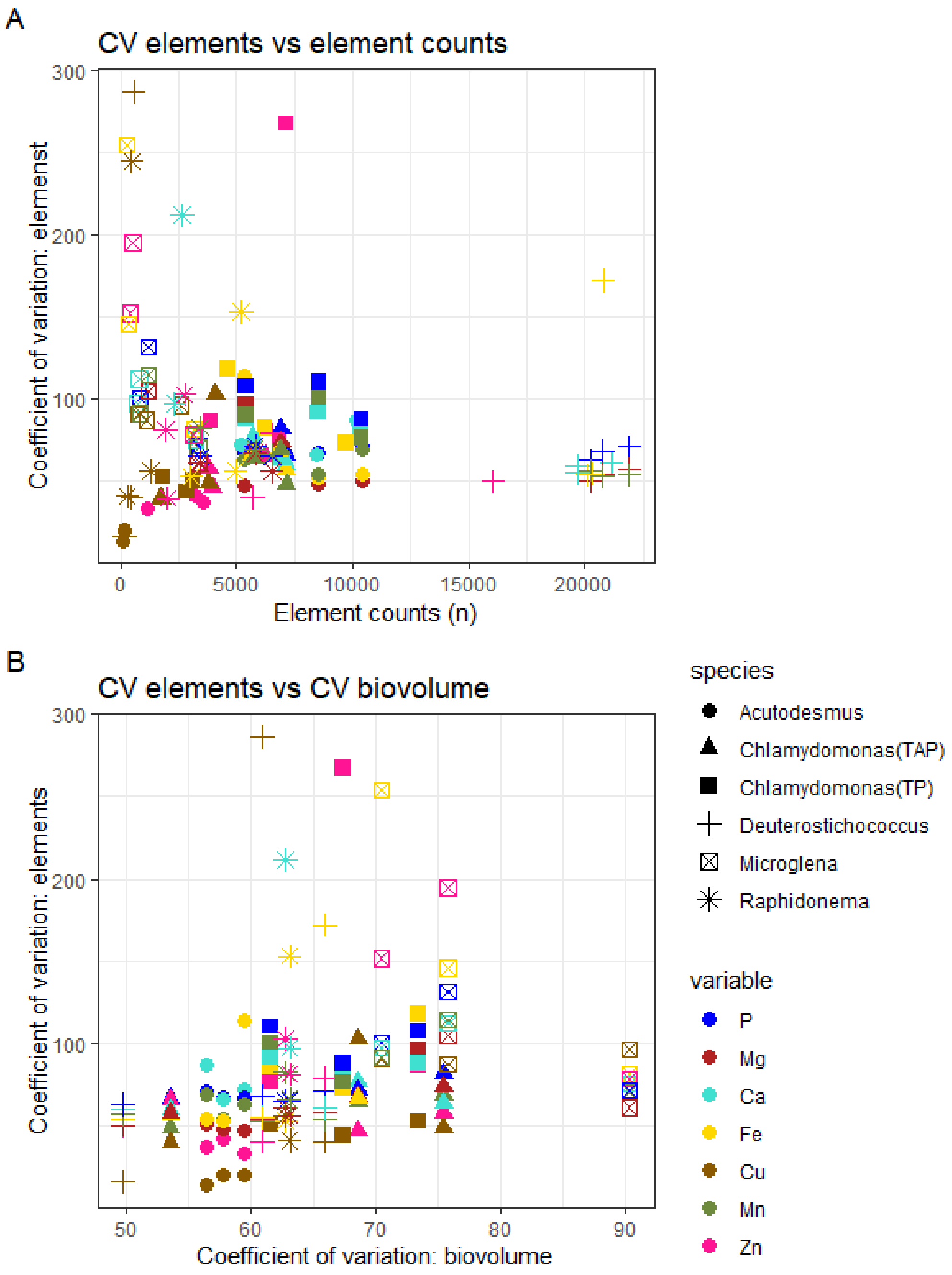

Supplementary Figure 2. Intra-sample elemental variation, measured by a coefficient of variation, compared to sample count number and sample biovolume variation. Coefficients of variation for the elemental masses of P, Mg, Ca, Cu, Fe, Mn and Zn plotted against the number of events measured **(A)** and against the coefficients of variation for cell biovolumes **(B)** for the cryosphere algae *Microglena sp.*, *Raphidonema Sempervirens, Deuterostichococus sp.,* and mesophilic species *Acutodesmus obliquus and Chlamydomonas reinhardtii* (grown in TAP and TP media).

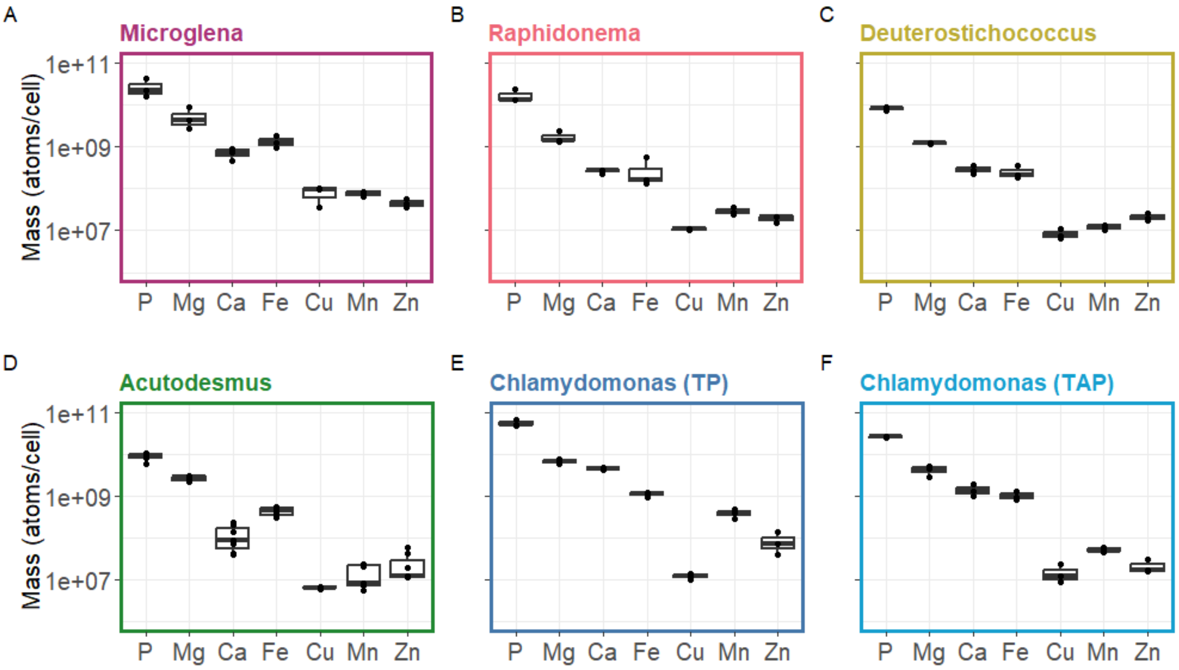

Supplementary Figure 3. Average mass (for each biological replicate) of each quantified element for each species plotted in atoms/cell (instead of fg/cell) from the single-cell ICP-ToF-MS data plotted in Figure 3. Cryosphere algae are **(A)** *Microglena sp.* (purple), **(B)** *Raphidonema sempervirens* (pink)*,* **(C)** *Deuterostichococus sp.* (yellow)*,* and mesophilic species are **(D)** *Acutodesmus obliquus* (green) *and Chlamydomonas reinhardtii* grown in **(E)** TP (dark blue) and **(F)** TAP media (light blue).

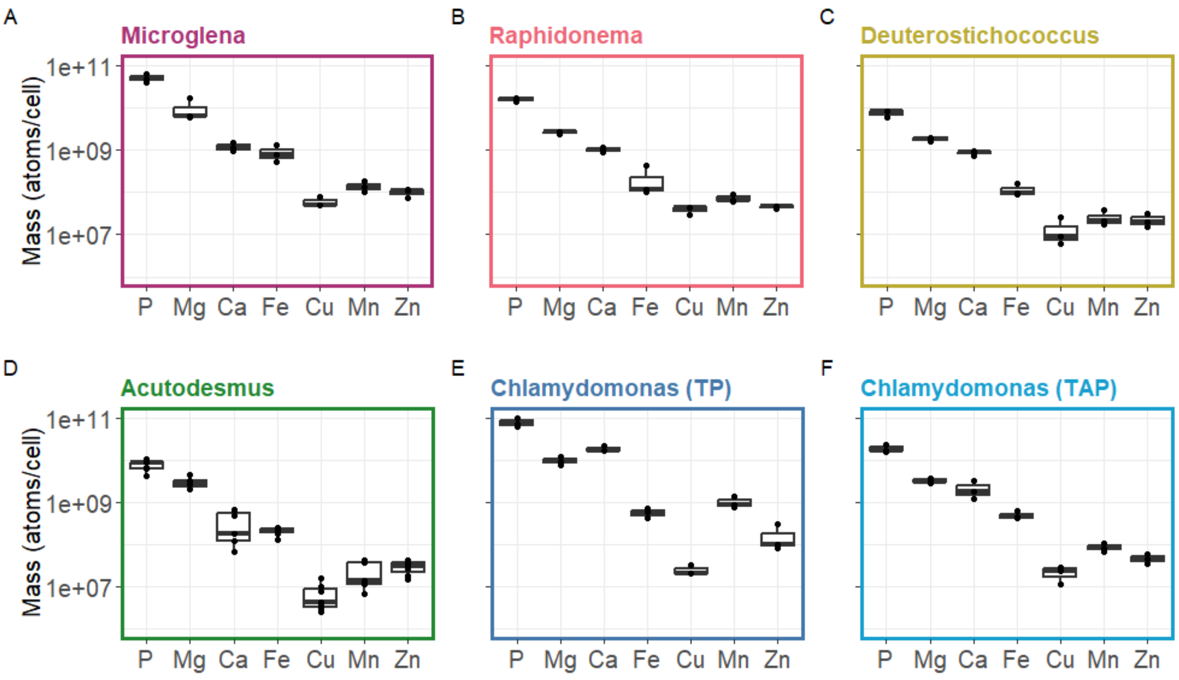

Supplementary Figure 4. Average (for each biological replicate) mass of each quantified element for each species plotted in atoms/cell (instead of fg/cell) from the digested data. Cryosphere algae are **(A)** *Microglena sp.* (purple), **(B)** *Raphidonema sempervirens* (pink), **(C)** *Deuterostichococcus sp.* (yellow), and mesophilic species are **(D)** *Acutodesmus obliquus* (green) *and Chlamydomonas reinhardtii* grown in **(E)** TP (dark blue) and **(F)** TAP media (light blue).
