# Supplementary material for: Single-cell ionomes of terrestrial cryosphere algae
Source: Commun Earth Environ. 2026 Jun 26;7(1):546. doi: 10.1038/s43247-026-03679-6 (PMC13309284; doi:10.1038/s43247-026-03679-6)
Supplement: Supplementary file 1 — Transparent Peer Review file [file 43247_2026_3679_MOESM1_ESM.pdf]

nature portfolio

Peer Review File

Single-cell ionomes of terrestrial cryosphere algae

Corresponding Author: Dr Silvana Oliveira

This file contains all editorial decision letters in order by version, followed by all author rebuttals in order by version.

Version 0:

Decision Letter:

** Please ensure you delete the link to your author home page in this e-mail if you wish to forward it to your coauthors **

Dear Dr Oliveira,

Your manuscript titled "Single cell ionomes of terrestrial cryosphere algae" has now been seen by 2 reviewers, and we include their comments at the end of this message. They find your work of interest, but some important points are raised. We are interested in the possibility of publishing your study in Communications Earth & Environment, but would like to consider your responses to these concerns and assess a revised manuscript before we make a final decision on publication.

We therefore invite you to revise and resubmit your manuscript, along with a point-by-point response that takes into account the points raised. Please highlight all changes in the manuscript text file.

Please submit your point-by-point responses as a separate file, distinct from your cover letter where you can add responses to the Editors' comments that you do not want to be made available to the reviewers. Word files are preferred. We recommend that any figures, tables or graphs that are included in the response to reviewers are also included in the main article or Supplementary Information.

We are committed to providing a fair and constructive peer-review process. Please don't hesitate to contact us if you wish to discuss the revision in more detail.

Please use the following link to submit your revised manuscript, point-by-point response to the referees' comments (which should be in a separate document to any cover letter), a tracked-changes version of the manuscript (as a PDF file) and the completed checklist:
Link Redacted
** This url links to your confidential home page and associated information about manuscripts you may have submitted or be reviewing for us. If you wish to forward this email to co-authors, please delete the link to your homepage first **

We hope to receive your revised paper within six weeks; please let us know if you aren't able to submit it within this time so that we can discuss how best to proceed. If we don't hear from you, and the revision process takes significantly longer, we may close your file. In this event, we will still be happy to reconsider your paper at a later date, as long as nothing similar has been accepted for publication at Communications Earth & Environment or published elsewhere in the meantime.

Please do not hesitate to contact us if you have any questions or would like to discuss these revisions further. We look forward to seeing the revised manuscript and thank you for the opportunity to review your work.

Best regards,

Jose Luis Iriarte Machuca, PhD
Editorial Board Member
Communications Earth & Environment

Somaparna Ghosh, PhD
Associate Editor - Communications Earth & Environment
Consulting Editor - Communications Sustainability

EDITORIAL POLICIES AND FORMATTING

We ask that you ensure your manuscript complies with our editorial policies. Please ensure that the following formatting requirements are met, and any checklist relevant to your research is completed and uploaded as a Related Manuscript file type with the revised article.

For Manuscripts that fall into the following fields:
• Behavioural and social science
• Ecological, evolutionary & environmental sciences
• Life sciences
An updated and completed version of our Reporting Summary must be uploaded with the revised manuscript
You can download the form here:
https://www.nature.com/documents/nr-reporting-summary.pdf

Furthermore, please align your manuscript with our format requirements, which are summarized on the following checklist:
Communications Earth & Environment formatting checklist

and also in our style and formatting guide Communications Earth & Environment formatting guide .

*** DATA: Communications Earth & Environment endorses the principles of the Enabling FAIR data project (http://www.copdess.org/enabling-fair-data-project/). We ask authors to make the data that support their conclusions available in permanent, publically accessible data repositories. (Please contact the editor if you are unable to make your data available).

All Communications Earth & Environment manuscripts must include a section titled "Data Availability" at the end of the Methods section or main text (if no Methods). More information on this policy, is available at http://www.nature.com/authors/policies/data/data-availability-statements-data-citations.pdf.

In particular, the Data availability statement should include:
- Unique identifiers (such as DOIs and hyperlinks for datasets in public repositories)
- Accession codes where appropriate
- If applicable, a statement regarding data available with restrictions
- If a dataset has a Digital Object Identifier (DOI) as its unique identifier, we strongly encourage including this in the Reference list and citing the dataset in the Data Availability Statement.

DATA SOURCES: All new data associated with the paper should be placed in a persistent repository where they can be freely and enduringly accessed. We recommend submitting the data to discipline-specific, community-recognized repositories, where possible and a list of recommended repositories is provided at http://www.nature.com/sdata/policies/repositories.

If a community resource is unavailable, data can be submitted to generalist repositories such as figshare or Dryad Digital Repository. Please provide a unique identifier for the data (for example a DOI or a permanent URL) in the data availability statement, if possible. If the repository does not provide identifiers, we encourage authors to supply the search terms that will return the data. For data that have been obtained from publically available sources, please provide a URL and the specific data product name in the data availability statement. Data with a DOI should be further cited in the methods reference section.

Please refer to our data policies at http://www.nature.com/authors/policies/availability.html.

REVIEWER COMMENTS:

Reviewer #1 (Remarks to the Author):

The study investigates ionome profiles of three chlorophyte algae species isolated polar environments (Microglena sp., Raphidonema sempervirens, and Deuterostichococus sp.) and performs a comparison with two mesophile chlorophyte algae (Acutodesmus obliquus and Chlamydomonas reinhardtii) using sc-ICP-ToF-MS. It is first study that shows that ionomes can be characterised in single cells in algae strains that were originally isolated from cryosphere environments. The single cell measurements were compared with bulk algae biomass.

The study evaluated effect of P-starvation on the elemental content of Raphidonema and Microglena who varied in their ionomes profiles grown in the absence of P in laboratory experiments.

Would it be possible to provide more ecologically informed justification for the selected growth temperature and light

conditions? Do the cryospheric algae have a psychrophilic physiology? Would it also be possible to consider in more details the differences in growth conditions for the cryospheric and mesophilic algae, and how the different growth conditions could have affected need and usage of trace metals and nutrients for growth; and to what extend it affects the comparability of the cryospheric and mesophilic algae.

It would also be an opportunity to consider if the use of psychrophilic instead of cryospheric would be more consistent terminology aligned with use of the term mesophilic algae.

It would also be important to add the conditions of the strains, e.g. axenic, clonal, unialgal, presence of bacteria and yeast in the methodologies as the conditions of the strains will have an effect on the results.

Reviewer #2 (Remarks to the Author):

Thanks for the opportunity to review “Single cell ionomes of terrestrial cryosphere algae” by Oliveira et al. The manuscript is very well written with thorough and detailed explanation, and well presented graphs and so forth.

The topic is certainly interesting, because these algae are becoming increasingly recognised for their impacts on globally important snow and ice habitats that are under the influence of climate change. It’s a great paper and I have only some minor questions when reading the text:

Small points:

Its described that there were not much differences between the three cryosphere algae and the two mesophilic strains (e.g. L501-504), although considering that these are each very different taxa grown in a few conditions, it might not be very meaningful to draw this direct comparison between mesophilic and cryophilic ionomes? In this reviewers opinion, many of the traits and features of algae are more specific to individual species.

Based on the introduction of the low temperature and high light as key variables in snow at the start of the manuscript, I did not fully follow why is P-starvation the key variable to study for these ionomes, as opposed to temperature, high light and so forth that might be more significant stress variables on snow and ice?

L254. sempervirens
L301,L305. Microglena
L419-421. the explanation that PERMANOVA test is too sensitive to enable identification of biological patterns seems an odd statistical explanation for the results?

** Visit Nature Portfolio's author and referees' website at www.nature.com/authors for information about policies, services and author benefits**

Communications Earth & Environment is committed to improving transparency in authorship. As part of our efforts in this direction, we are now requesting that all authors identified as ‘corresponding author’ create and link their Open Researcher and Contributor Identifier (ORCID) with their account on the Manuscript Tracking System prior to acceptance. ORCID helps the scientific community achieve unambiguous attribution of all scholarly contributions. You can create and link your ORCID from the home page of the Manuscript Tracking System by clicking on ‘Modify my Springer Nature account’ and following the instructions in the link below. Please also inform all co-authors that they can add their ORCIDs to their accounts and that they must do so prior to acceptance.
https://www.springernature.com/gp/researchers/orcid/orcid-for-nature-research

For more information please visit http://www.springernature.com/orcid

If you experience problems in linking your ORCID, please contact the Platform Support Helpdesk.

Version 1:

Decision Letter:

** Please ensure you delete the link to your author home page in this e-mail if you wish to forward it to your coauthors **

Dear Dr Oliveira,

Your manuscript titled "Single-cell ionomes of terrestrial cryosphere algae" has now been seen by our reviewers, whose comments appear below. In light of their advice we are delighted to say that we are happy, in principle, to publish a suitably revised version in Communications Earth & Environment.

We therefore invite you to revise your paper one last time to address the remaining concerns of our reviewers. At the same time we ask that you edit your manuscript to comply with our format requirements and to maximise the accessibility and therefore the impact of your work.

EDITORIAL REQUESTS:

Please review our specific editorial comments and requests regarding your manuscript in the attached "Editorial Requests Table".

*****Please take care to match our formatting and policy requirements. We will check revised manuscript and return manuscripts that do not comply. Such requests will lead to delays. *****

Please outline your response to each request in the right hand column. Please upload the completed table with your manuscript files as a Related Manuscript file.

If you have any questions or concerns about any of our requests, please do not hesitate to contact me.

SUBMISSION INFORMATION:

In order to accept your paper, we require the files listed at the end of the Editorial Requests Table; the list of required files is also available at https://www.nature.com/documents/commsj-file-checklist.pdf .

OPEN ACCESS:

Communications Earth & Environment is a fully open access journal. Articles are made freely accessible on publication. For further information about article processing charges, open access funding, and advice and support from Nature Portfolio, please visit https://www.nature.com/commsenv/open-access

At acceptance, you will be provided with instructions for completing the open access licence agreement on behalf of all authors. This grants us the necessary permissions to publish your paper. Additionally, you will be asked to declare that all required third party permissions have been obtained, and to provide billing information in order to pay the article-processing charge (APC).

Please use the following link to submit the above items:
Link Redacted
** This url links to your confidential home page and associated information about manuscripts you may have submitted or be reviewing for us. If you wish to forward this email to co-authors, please delete the link to your homepage first **

We hope to hear from you within two weeks; please let us know if you need more time.

Best regards,

Somaparna Ghosh, PhD
Associate Editor,
Communications Earth & Environment
Consulting Editor,
Communications Sustainability

REVIEWERS' COMMENTS:

Reviewer #1 (Comments to the Authors): Reviewer #1 has informed us they are happy with the revised version.

** Visit Nature Portfolio's author and referees' website at www.nature.com/authors for information about policies, services and author benefits**

Open Access This Peer Review File is licensed under a Creative Commons Attribution 4.0 International License, which permits use, sharing, adaptation, distribution and reproduction in any medium or format, as long as you give appropriate credit to the original author(s) and the source, provide a link to the Creative Commons license, and indicate if changes were made.
In cases where reviewers are anonymous, credit should be given to 'Anonymous Referee' and the source.
The images or other third party material in this Peer Review File are included in the article's Creative Commons license, unless indicated otherwise in a credit line to the material. If material is not included in the article's Creative Commons license and your intended use is not permitted by statutory regulation or exceeds the permitted use, you will need to obtain permission directly from the copyright holder.
To view a copy of this license, visit https://creativecommons.org/licenses/by/4.0/

Point-by-point response to the referees' comments

The authors would like to thank the reviewers for their valuable comments, which enabled us to critically re-examine and improve the manuscript.

Changes are highlighted in yellow in the revised manuscript.

Reviewer #1 (Remarks to the Author):

The study investigates ionome profiles of three chlorophyte algae species isolated polar environments (Microglena sp., Raphidonema sempervirens, and Deuterostichococcus sp.) and performs a comparison with two mesophile chlorophyte algae (Acutodesmus obliquus and Chlamydomonas reinhardtii) using sc-ICP-ToF-MS. It is first study that shows that ionomes can be characterised in single cells in algae strains that were originally isolated from cryosphere environments. The single cell measurements were compared with bulk algae biomass.

The study evaluated effect of P-starvation on the elemental content of Raphidonema and Microglena who varied in their ionomes profiles grown in the absence of P in laboratory experiments.

Would it be possible to provide more ecologically informed justification for the selected growth temperature and light conditions?

Regarding the justification of growth temperature, light conditions, as well as the nutrients in the media used for the different cultures, the justification is linked more to the standard culture conditions that are used for the taxa (in our laboratory and elsewhere), rather than ecological conditions.

Regarding the temperature for the cryosphere algae, we used the growth temperature that is standard in our laboratory (4°C). This temperature (or within this range) has been used in other research of cryosphere algae (Broadwell *et al.* 2023, Jensen *et al.* 2023). This cold temperature (a little above 0) was chosen in order to represent the temperatures found in melting snow packs.

Regarding the light conditions, we specifically grew the cryosphere cultures under low light conditions (~50 µmol/m2/s, which are the standard light conditions that we use in our laboratory). Based on data from our laboratory (data not shown as will be part of a separate manuscript), these light conditions are low stress, in particular for *Raphidonema* (described as light sensitive by CCCryo).

We have updated the method with extra information in this section (page 4, lines 146-149):

"Cryosphere algae cultures were grown in a temperature (at 4°C to mimic the temperatures of melting snow packs and glacier surfaces) and light controlled (~50 µmol/m2/s, at 16h/8h light/dark conditions) incubator (Percival, USA). The light conditions were chosen to reduce any effect of high light stress on the cryosphere algal taxa (data not shown), as light stress was not an object of study in our study."

Do the cryospheric algae have a psychrophilic physiology?

We thank the reviewer for this valuable question. We have added this information to the method section with references (page 4, lines 144-145):

"Microglena sp. and Raphidonema sempervirens are both psychrophilic algae (as described by CCCryo), while Deuterostichococcus sp. is psychrotolerant)."

Would it also be possible to consider in more details the differences in growth conditions for the cryospheric and mesophilic algae, and how the different growth conditions could have affected need and usage of trace metals and nutrients for growth; and to what extend it affects the comparability of the cryospheric and mesophilic algae.

This is a very relevant point regarding the comparability of the taxa that we are studying. We previously had this sentence to address this topic (page 19, lines 505-511):

"P, Mg, and Ca exist in cells in relatively high abundance to maintain a variety of cellular metabolic pathways, such as energy transfer, nucleic acid synthesis, photosynthesis and signalling69–72. Life in the cold will induce lower metabolic rates73, which could underpin a lowered cellular requirement for P, Mg and Ca. Trace metal-wise, we were not able to detect an important trend other than a high calculated C:Zn ratio in Microglena and Raphidonema, suggesting that this element may not be as biologically important for these taxa compared to the other cells analysed."

And we have added the following sentences to this paragraph to provide more insight (page 5, lines 525-530):

Our data indicates that, except for a potential role for Zn in Microglena and Raphidonema, trace metal cellular content (and potential associated cellular trace metal use), does not appear to be impacted by temperature. Why cellular trace metals, who are essential co-factors in cellular metabolism, is not impacted by temperature despite temperature slowing metabolism is not clear. Further work combining ionomics with other omics work such as genomics and proteomics is necessary to fully assess trace metal homeostasis in algae isolated from the terrestrial cryosphere.

We also added this sentence to the start of the paragraph (page 19, lines 517-518):

"However, the growth temperature used for the cryosphere algae compared to the mesophilic taxa likely impacted the ionomes of the taxa studied."

It would also be an opportunity to consider if the use of psychrophilic instead of cryospheric would be more consistent terminology aligned with use of the term mesophilic algae.

This is a good point, however as the cryospheric algae are not all psychrophilic, this is not possible. The terms "cryospheric" or "cryosphere" as an adjective is used throughout as an umbrella term for algae living in the terrestrial cryosphere, as the three studied strains have been isolated from (and can live on) different substrates in the cryosphere. Indeed, they would normally be distinguished by their growth substrate (snow, glacier ice, and/or soil). It is therefore a helpful umbrella term for all the no-mesophile algae studied, regardless of their ideal growth substrate and cellular adaptation to life in the cold.

To address this comment, we have removed all "cryospheric" from the text, referring to them only as "cryosphere algae" or "algae isolated from the terrestrial cryosphere". This allows them to be distinguished from the mesophilic taxa as algae who have been isolated from a cold environment.

It would also be important to add the conditions of the strains, e.g. axenic, clonal, unialgal, presence of bacteria and yeast in the methodologies as the conditions of the strains will have an effect on the results.

We thank the reviewer for this suggestion. This information has now been added to the methods section of the manuscript (page 4, lines 140-144):

"All cultures were unialgal. Microglena sp., Chlamydomonas reinhardtii, and Acutodesmus obliquus were all axenic cultures. Raphidonema sempervirens (as described by CCCryo) and Deuterostichococcus sp. were non-axenic cultures. However, for all analyzed samples, we did not find visible bacterial cells (checked by microscopy and by centrifugation)."

We have not added information regarding fungal presence (there was no fungal presence) or if they were clonal (the information is partially missing, and we do not think it is necessarily relevant for this study).

Reviewer #2 (Remarks to the Author):

Thanks for the opportunity to review "Single cell ionomes of terrestrial cryosphere algae" by Oliveira et al. The manuscript is very well written with thorough and detailed explanation, and well presented graphs and so forth.

We thank the reviewer for their kind remarks.

The topic is certainly interesting, because these algae are becoming increasingly recognised for their impacts on globally important snow and ice habitats that are under the influence of climate change. It's a great paper and I have only some minor questions when reading the text:

Small points:

Its described that there were not much differences between the three cryosphere algae and the two mesophilic strains (e.g. L501-504), although considering that these are each very different taxa grown in a few conditions, it might not be very meaningful to draw this direct comparison between mesophilic and cryophilic ionomes? In this reviewers opinion, many of the traits and features of algae are more specific to individual species.

We thank the reviewer for their input here. We have added the following two sentences to the discussion (pages 18 and 19, lines 511-516):

"For both these algal categories presented here, it is therefore a possibility that the difference in taxonomy (and the associated evolutionary history) supersedes habitat conditions in terms of their influence on ionomes under nutrient-replete laboratory culture conditions. Indeed, under such conditions, different evolutionary histories (for example, belonging to the Trebouxiophyceae vs. the Chlorophyceae class) as well as species-specific differences, could influence cellular ionomes more than the habitat they came from."

Based on the introduction of the low temperature and high light as key variables in snow at the start of the manuscript, I did not fully follow why is P-starvation the key variable to study for these ionomes, as opposed to temperature, high light and so forth that might be more significant stress variables on snow and ice?

We apologize for the confusion with the justification of this experiment. Globally, we undertook the P-starvation work to assess the robustness of quantifying single-cell ionomes within biological experiments (as explained in the first paragraph of the last results section, page 15, lines 413-416). Phosphorus limitation stress is well studied across algal taxa (as described in the discussion) and thus could form a good starting point to look at the effect of abiotic stress on cryosphere taxa using single-cell ionomes. Temperature and light stress

are indeed also very important to study and should be part of further study. The conclusion already contains the following sentence (page 20, lines 579-583):

Cellular nutrient status will not only be a by-product of nutrient availability, but also of external factors such as temperature which will impact metabolic rates, liquid water availability which will impact primary productivity (thus likely altering cellular Mg content), and light stress which will affect oxidative stress pathways (thus likely increasing the cellular requirements for trace metals such as Fe, Cu, and Mn).

We have also added the following sentence to the conclusion (page 20, lines 583-585):

"Investigating the impact of light and temperature (including freezing) stress on terrestrial cryosphere algae ionomes is an important future research goal."

However, nutrient stress is also generally considered an important abiotic stressor as described in the last section of the first introduction paragraph. Understanding how cryosphere algal taxa use and store nutrients, and how they respond to very variable environmental nutrient concentrations is important. Therefore, quantifying the impact of P-limitation on algal ionomes is relevant for our understanding of taxa from this environment. The references to justify for N and P limitation in the terrestrial cryosphere are cited at the start of the second paragraph of the introduction. We have added the following sentence to the last paragraph of the discussion with references (page 20, lines 561-562):

"P concentrations in terrestrial cryosphere environments are known to be low and/or limiting for biota, including algae."

L254. Sempervirens ??
L301,L305. Microglena ??

Both of the above have been updated.

L419-421. the explanation that PERMANOVA test is too sensitive to enable identification of biological patterns seems an odd statistical explanation for the results?

We thank the reviewer for this valuable observation. We have removed this explanation from the results. Instead, we have updated the discussion section on these results to read (page 19, lines 537-539)

"*P starvation in Microglena led to reduced cellular P and Mg and increased Cu, with no reproducible (across biological replicates and statistical analysis types) important change for Raphidonema (Fig. 7-8)".*

Additional changes:

A couple of references have also been updated and are in yellow.

All figures and references to *Deuterostichococus* have now been updated to *Deuterostichococcus.*

All the "single cell" terms in the manuscript were updated to the corrected form (as an adjective) as "single-cell".

Point-by-point response to the referees' comments #2

Reviewer #1 (Comments to the Author):

Reviewer #1 has informed us they are happy with the revised version.

We thank Reviewer #1 for their valuable comments and remarks during the revision process and for revising our manuscript once again.
